# Targeting E2F8 sensitizes gemcitabine-resistant gallbladder cancer to PARP inhibitors by disrupting RRM2-driven DNA repair

**DOI:** 10.1186/s13046-025-03586-2

**Published:** 2025-12-14

**Authors:** Xiaojian Ni, Min Li, Wenqing Qiu, Jichang Han, Meng Yu, Shulong Zhang, Bohao Zheng, Jiaqi He, Houbao Liu

**Affiliations:** 1 Department of Biliary Surgery, Zhongshan Hospital, Fudan University, Shanghai, 200030 China; 2 Department of General Surgery, Shanghai Xuhui Central Hospital, Shanghai, 200031 China; 3 Zhongshan-Xuhui Hospital, Fudan University, Shanghai, 200032 China; 4 Biliary Tract Disease Center of Zhongshan Hospital, Fudan University, Shanghai, 200030 China; 5 Cancer Center, Zhongshan Hospital, Fudan University, Shanghai, 200030 China; 6 Shanghai Biliary Tract Minimal Invasive Surgery and Materials Engineering Research Center, Shanghai, 200032 China; 7 School of Life Science, Hangzhou Institute for Advanced Study, University of Chinese Academy of Sciences, Hangzhou, 310024 China; 8 Sylvester Comprehensive Cancer Center, University of Miami, Miller Scholl of Medicine, Miami, FL 33136 USA; 9 General Surgery, Department of Huadong Hospital, Fudan University, Shanghai, 200040 China

**Keywords:** Gallbladder cancer, Gemcitabine resistance, E2F8, RRM2, PARP inhibitor, DNA damage repair, Patient-derived organoids

## Abstract

**Background:**

Gallbladder cancer (GBC) is an aggressive malignancy with limited therapeutic options, primarily due to the frequent emergence of resistance to gemcitabine-based chemotherapy. Uncovering molecular mechanisms underlying this resistance is essential for developing more effective treatments.

**Methods:**

Gemcitabine-resistant GBC cell lines were generated and subjected to transcriptomic sequencing to identify resistance-associated genes. A genome-wide CRISPR-Cas9 knockout screen was used to pinpoint key genetic regulators. Functional validation was performed through gene knockdown and overexpression, cell viability and apoptosis assays, colony formation, and DNA damage analysis. A high-throughput virtual screening (HTVS) approach was applied to identify small-molecule inhibitors targeting the E2F8-DNA interaction. The efficacy of selected compounds was tested in vitro and in xenograft mouse models, and further validated using patient-derived organoids (PDOs) established from primary and recurrent gallbladder cancers.

**Results:**

The transcription factor E2F8 was identified as a driver of gemcitabine resistance via upregulation of RRM2, a gene involved in DNA repair. Knockdown of E2F8 enhanced sensitivity to poly (ADP-ribose) polymerase (PARP) inhibitors in resistant GBC cells by impairing DNA repair. HTVS yielded HIT-4, a small-molecule inhibitor that binds to E2F8 and disrupts its interaction with DNA, leading to reduced RRM2 expression. HIT-4 significantly increased apoptosis and DNA damage when combined with PARP inhibitors. In vivo and in PDO models, HIT-4 and PARP inhibitor co-treatment markedly suppressed tumor growth, extended survival, and showed minimal toxicity.

**Conclusions:**

This study identifies the E2F8-RRM2 axis as a key regulator of gemcitabine resistance in GBC and establishes E2F8 as a druggable target. The novel compound HIT-4, in combination with PARP inhibitors, represents a promising therapeutic strategy to overcome chemoresistance and warrants further clinical investigation.

**Supplementary Information:**

The online version contains supplementary material available at 10.1186/s13046-025-03586-2.

## Introduction

Gallbladder cancer (GBC) is an aggressive and highly lethal malignancy, accounting for 80–95% of biliary tract cancers globally, with incidence rates continuing to rise [1, 2]. Due to its typically asymptomatic nature in early stages, most patients are diagnosed at advanced stages, resulting in a five-year survival rate of less than 10% [3]. Surgery resection remains the primary treatment; however, most patients require adjunctive chemotherapy, with gemcitabine-based regimens currently constituting the standard first-line treatment [4, 5]. Unfortunately, intrinsic or acquired chemoresistance severely limits the effectiveness of these treatments, leading to dismal patient outcomes and underscoring an urgent clinical need for novel therapeutic strategies targeting gemcitabine resistance in GBC.

Poly (ADP-ribose) polymerase (PARP) inhibitors function by disrupting DNA single-strand break repair, thereby inducing synthetic lethality specifically in tumors deficient in homologous recombination repair pathway [6, 7]. Preclinical and clinical investigations have demonstrated promising efficacy of PARP inhibitors across multiple malignancies, including ovarian, breast, and prostate cancers harboring mutations in *BRCA1/2* or other DNA damage response (DDR)-related genes [8–10]. Additionally, PARP inhibitors have been explored extensively as chemosensitizing agents, enhancing treatment response in various drug-resistant cancers [11]. Whole-genome CRISPR-Cas9 screening has emerged as a robust method to systematically uncover genetic vulnerabilities and mechanisms underlying drug resistance, guiding the development of targeted therapies for chemotherapy-resistant cancers [12, 13]. Although such screens have identified genetic determinants critical to PARP inhibitor sensitivity in prostate, lung, and ovarian cancers [14–16], their application in the context of GBC has remained limited [17], with few studies or clinical trials specifically addressing this malignancy.

E2F transcription factor 8 (E2F8) plays pivotal role in cell cycle regulation, cellular proliferation, and the DNA damage response [18]. Dysregulated expression of E2F8 has been implicated in tumor progression and therapeutic resistance across various cancers, highlighting its potential as a therapeutic target [19–23]. A critical downstream effector of E2F8, ribonucleotide reductase regulatory subunit M2 (RRM2), is essential for DNA synthesis and repair, thereby contributing significantly to genomic stability [24, 25]. Elevated RRM2 levels have been consistently associated with poor prognosis and increased resistance to chemotherapy in diverse cancer types [26–28].

To investigate mechanisms underlying gemcitabine resistance in GBC, we established gemcitabine-resistant gallbladder cancer cell lines and conducted transcriptomic sequencing to identify key genetic alterations linked to resistance. Utilizing whole-genome CRISPR-Cas9 screening, we revealed that knockout of E2F8 significantly enhanced sensitivity to PARP inhibitors in gemcitabine-resistant cells. Mechanistically, E2F8 promote DNA repair by transcriptionally regulating RRM2 expression; inhibition of E2F8 consequently results in increased DNA damage accumulation. Furthermore, we identified and validated a novel small molecule inhibitor targeting E2F8 via high-throughput virtual screening, demonstrating effective suppression of RRM2 expression. Importantly, combining this small molecule with PARP inhibitors exhibited potent therapeutic efficacy, providing a promising strategy for overcoming gemcitabine resistance in gallbladder cancer.

## Materials and methods

### Cell culture and reagents

The human gallbladder cancer cell line GBS-SD was obtained from the Type Culture Collection of the Chinese Academy of Science (Shanghai, China), NOZ cell line was purchased from the Health Science Research Resources Bank (Osaka, Japan). Both cell lines were cultured in DMEM medium supplemented with 10% fetal bovine serum (FBS). Daoy and D341 (medulloblastoma), Capan-1 (pancreatic), UWB1.289 (ovarian), and UACC-893 (breast) cells were obtained from ATCC and cultured in MEM medium containing 10% FBS. Human cancer cell lines SW1353, CACO2, and MDA-MB-231 were acquired from the Stem Cell Bank, Chinese Academy of Sciences, and maintained in DMEM medium with 10% FBS. All cell lines were routinely tested and confirmed to be free from mycoplasma contamination and cultured at 37℃ in a humidified incubator with 5% CO_2_.

#### Reagents

Gemcitabine (HY-B0003) and Olaparib (HY-10162) were purchased from MedChemExpress. HIT-3 (#8003 − 1541), HIT-4 (#2036 − 0727), and HIT-7 (#8009–8689) were obtained from ChemDiv.

#### Antibodies

Rabbit monoclonal antibodies against E2F8 (#346661), RRM2 (#65939), and GAPDH (#2118) were purchased from Cell Signaling Technology. Rabbit monoclonal anti-H2AX antibody (ab229914) and mouse monoclonal anti-beta-actin antibody (ab8226) were sourced from Abcam. Mouse monoclonal anti-phospho-H2A.X (Ser139) antibody (#05–636) was obtained from Millipore Sigma.

### High-throughput virtual screening

The X-ray crystal structure of human E2F8 (PDB ID: 4YO2) was obtained from the Protein Data Bank and processed using the Protein Preparation Wizard module in Schrödinger (version 11.4). Preparation steps included the addition of hydrogen atoms, assignment of bond orders, filling in missing side chains, and energy minimization. The potential ligand-binding site was identified using the SiteMap module and defined based on the DNA-binding cavity, encompassing key residues (A113, A115–118, A120–121, A124, A149–152, A156, A317, A320). A receptor grid box centered on this site was generated with dimensions set of 20 Å × 20 Å × 20 Å. Two commercially available compound libraries, ChemDiv (D001) and TargetMol (T001), were curated for virtual screening. The 2D structures were converted to energy-minimized 3D conformers using the LigPrep module, which accounted for protonation states, tautomer generation, and geometry optimization.

High-throughput virtual screening was performed using the Virtual Screening Workflow in Schrödinger. Initial docking was carried out in Glide-HTVS mode. The top 10% of hits were subsequently redocked using Glide-SP mode, followed by further refinement of the top 10% using Glide-XP. Compounds were filtered based on drug-likeness using the quantitative estimate of drug-likeness (QED), with a cutoff of QED scores > 0.3. Molecules containing pan-assay interference structure (PAINS) motifs were excluded to minimize false positives.

### Genome-wide CRISPR-Cas9 knockout screening

A genome-wide CRISPR-Cas9 loss-of-function screen was performed using the human GeCKO v2 pooled sgRNA library (Addgene, #52961), which encodes both sgRNAs and Cas9 in a single lentiviral construct. The library comprises two sub-libraries, A and B, each containing 3 sgRNAs per gene and 1,000 non-targeting control sgRNAs. To optimize infection efficiency and multiplicity of infection (MOI), preliminary lentiviral transductions were conducted in NOZ-R cells using an eGFP-expressing control virus. Cells were infected with serial dilutions of virus (MOI = 0.3, 0.5, 1, 2) in the presence of 5 µg/mL polybrene, followed by centrifugation at 2,000 rpm for 2 h and incubation for 6 h before replacing the medium. Transduction efficiency was assessed 48 h later via FACS. An MOI of < 0.5 was selected to maximize single sgRNA integration per cell.

For the screening experiment, 1.8 × 10⁸ NOZ-R cells were seeded across ten 15-cm dishes to ensure at least 400-fold coverage of the 123,411 unique sgRNAs. Lentiviral infection was followed by puromycin selection (2 µg/mL) for 7 days. After selection, cells were divided into two groups: a control group treated with DMSO, and an experimental group treated with sublethal doses of olaparib for 14 days. Surviving cells were expanded and harvested for genomic DNA extraction. Genomic DNA was isolated using standard protocols, and sgRNA cassettes were amplified via PCR using primers targeting the lentiCRISPR v2 vector backbone. Amplicons were purified, followed by end-repair, A-tailing, and adapter ligation for NGS library preparation. Differential sgRNA representation between DMSO- and Olaparib-treated samples was analyzed to identify sgRNA significantly enriched or depleted, indicative of genes associated with drug resistance or sensitization. Gene-level enrichment analysis was performed using MAGeCK algorithms, and functional annotation was conducted using KEGG pathway databases.

### Establishment of gemcitabine-resistant cell lines and RNA sequencing

To generate gemcitabine-resistant gallbladder cancer cell lines, parental NOZ and GBC-SD cells were continuously cultured in the presence of stepwise-increasing concentrations of gemcitabine (HY-B0003, MedChemExpress). The selection process began with a sublethal concentration of 10 nM, under which cells were maintained until they resumed stable proliferation. The gemcitabine concentration was subsequently increased in two-fold increments every 2–3 passages, reaching a final concentration of 1 µM over the course of approximately six months. The resulting resistant sublines (designated NOZ-R and GBC-SD-R) were maintained in 1 µM gemcitabine. For all downstream experiments, cells were cultured in drug-free medium for at least one week to eliminate drug effects.

Total RNA was extracted from both parental and resistant cell lines using TRIzol reagent according to the protocol. RNA integrity and concentration were assessed using an Agilent 2100 Bioanalyzer. Sample with RNA Integrity Number (RIN) ≥ 7.0 were sent to Majorbio (Shanghai, China) for transcriptome profiling by next-generation RNA sequencing (RNA-seq). Raw sequencing data underwent standard quality control, alignment to the human reference genome (GRCh38), and differential gene expression analysis.

### Protein purification, and Surface Plasmon Resonance (SPR) assay

The coding sequence of the human E2F8 DNA-binding domain (residues Gln_110_–Ile_350_), including both the wild-type and the R156A/R314A mutant variants, was cloned into the pETG-20 A expression vector containing an N-terminal thioredoxin and 6 × His tag. Expression was carried out in E. coli Rosetta (DE3) pLysS cells as previously described [29].

Bacterial pellets were lysed in immobilized metal affinity chromatography (IMAC) lysis buffer (50 mM Tris-HCl, pH 7.5, 300 mM NaCl, 10 mM imidazole, 10% glycerol). Clarified lysates were loaded onto a HisTrap HP column pre-equilibrated with buffer containing 100 mM HEPES, pH 7.5, 500 mM NaCl, 10 mM imidazole, 10% glycerol, and 0.5 mM TCEP. Bound proteins were eluted with buffer containing 500 mM imidazole. The His-thioredoxin tag was removed by TEV protease digestion overnight at 4 °C, followed by further purification using size-exclusion chromatography on a HiLoad 16/600 Superdex 200 column equilibrated with 20 mM HEPES, pH 7.5, 150 mM NaCl, 5% glycerol, and 0.5 mM TCEP. Protein purity and integrity were confirmed by SDS–PAGE.

SPR experiments were performed on a Biacore 8 K system at 25 °C. Wild-type and mutant E2F8 proteins were diluted to 200 µg/mL in 10 mM sodium acetate buffer (pH 4.5) and immobilized onto CM5 sensor chips via standard amine coupling, yielding approximately 10,000 response units (RU) per flow cell. Small-molecule compounds HIT-3, HIT-4, and HIT-7 were prepared in running buffer (20 mM HEPES, pH 7.5, 200 mM NaCl, 1% DMSO) and injected at concentrations ranging from 39.0625 nM to 25 µM using a series of two-fold dilutions. Association and dissociation phases were monitored for 180 s and 330 s, respectively, at a flow rate of 30 µL/min. Data were analyzed using Biacore Insight Evaluation Software, and binding kinetics were calculated using a 1:1 steady-state affinity model.

### Microscale Thermophoresis (MST) assay

The binding affinity between E2F8 and the small molecule HIT-4 was assessed using microscale thermophoresis (MST), as previously described [30]. Recombinant wild-type E2F8 DNA-binding domain was labeled with the RED-tris-NTA dye according to the manufacturer’s instructions. Briefly, E2F8 was diluted to 10 µM in HEPES buffer and incubated with the dye for 30 min at room temperature in the dark. Labeled protein was purified via desalting columns and adjusted to a final concentration of 2 µM. A 16-point 1:1 serial dilution of HIT-4 was prepared in HEPES buffer containing 0.1% DMSO, with concentration ranging from 50 µM to 1.53 nM. Equal volumes (10 µL) of labeled E2F8 and HIT-4 dilutions were mixed, yielding final concentrations of 1 µM E2F8 and 25 µM to 0.76 nM HIT-4. The mixtures were incubated at room temperature for 10 min before loading into standard Monolith NT.115 capillaries. MST measurements were performed using a Monolith NT.115 instrument at 25 °C with 80% excitation power and 40% MST power. All measurements were performed in triplicate. Normalized fluorescence (Fnorm) values were plotted against HIT-4 concentrations, and the dissociation constant (Kd) was calculated using MO.Affinity Analysis software.

### Chromatin Immunoprecipitation (ChIP) and quantitative PCR (qPCR)

Chromatin immunoprecipitation was performed using the SimpleChIP Enzymatic Chromatin IP Kit (CST, #9003) following the manufacturer’s instructions. NOZ-R and GBC-SD-R cells were treated with either DMSO or HIT-4 (1 µM) for 6 h, followed by fixation with 1% formaldehyde for 10 min at room temperature. Crosslinking was quenched with 125 mM glycine, and cells were lysed, and subjected to chromatin digestion using micrococcal nuclease. Digested chromatin was further sheared by brief sonication to generate fragments ranging from 150 to 900 bp. Chromatin samples were incubated overnight at 4 °C with anti-E2F8 antibody, and immune complexes were captured using protein G magnetic beads. After a series of washes, chromatin complexes were eluted, and crosslinks were reversed by overnight incubation at 65 °C. DNA was purified using spin columns provided in the kit.

To evaluate E2F8 binding at the *RRM2* promoter, four candidate binding sites were predicted using the JASPAR online tool (http://jaspar.genereg.net), and specific primers were designed to amplify each site. An unrelated genomic region within the *RRM2* locus was selected as a negative control. Quantitative PCR (qPCR) was performed using SYBR Green Master Mix on a QuantStudio real-time PCR system. ChIP-qPCR results were normalized to input DNA and presented as fold enrichment relative to IgG control.

### Cell viability, apoptosis, and colony formation assay

Cell viability was assessed using the CellTiter-Glo Luminescent Cell Viability Assay (Promega) following the manufacturer’s instructions. For drug sensitivity analysis, NOZ, NOZ-R, GBC-SD, and GBC-SD-R cells were seeded in 96-well clear-bottom plates (Costar, Cat. #3160) and treated the following day with serial dilutions of gemcitabine. After 5 days of incubation, luminescence was measured using a microplate reader (BioTek Synergy). In separate experiments, NOZ-R and GBC-SD-R cells were transfected with sgRNAs or shRNAs targeting E2F8, followed by treatment with either DMSO or varying concentrations of Olaparib. Cell viability was measured on day 5 post-treatment. To assess potential synergy, cells were treated with increasing concentrations of Olaparib alone or in combination with HIT-4 (1 µM), and viability was similarly measured after 5 days. Sensitivity to HIT-4 alone was also tested in multiple cancer cell lines including CACO-2, MDA-MB-231, Daoy, SW1353, UACC-893, and D341.

Apoptosis was evaluated using the Annexin V-FITC/PI Apoptosis Detection Kit (BD Biosciences, Cat. #556547). NOZ-R and GBC-SD-R cells stably expressing control shRNA or E2F8 shRNA were treated with DMSO, Olaparib, HIT-4, or a combination of both compounds. After 72 h, cells were harvested, washed with PBS, and stained with FITC-conjugated Annexin V and propidium iodide for 15 min at room temperature. Samples were analyzed using a CytoFLEX flow cytometer (Beckman Coulter), and data were processed with FlowJo software (BD Biosciences).

Colony formation assays were performed to assess long-term proliferative capacity. NOZ-R and GBC-SD-R cells, including those stably expressing E2F8 shRNA, E2F8 overexpression, or RRM2 overexpressing constructs, were seeded in 6-well plates and treated as indicated. In drug treatment experiments, NOZ-R, GBC-SD-R, SW1353, and D341 cells were exposed to DMSO, HIT-4, Olaparib, or their combination for 14 days. Colonies were fixed with 4% paraformaldehyde for 15 min, stained with 0.5% crystal violet solution (Beyotime, Cat. #C0121), washed, and air-dried before imaging. Colony numbers were quantified using ImageJ. All experiments were performed in biological triplicates.

### Immunofluorescence and quantification of γ-H2AX foci formation

To evaluate DNA damage response, immunofluorescence staining for γ-H2AX foci was performed in NOZ, NOZ-R, GBC-SD, and GBC-SD-R cells under various treatment conditions. For gemcitabine-induced DNA damage, cells were seeded on sterile glass coverslips in 24-well plates and treated with gemcitabine for 30 min. After drug removal via PBS wash, cells were incubated in fresh medium for 4 h and then fixed with 4% paraformaldehyde for 15 min at room temperature.

For Olaparib treatment, NOZ-R and GBC-SD-R cells stably expressing shE2F8 or RRM2 overexpression constructs were treated with Olaparib (10 µM) for 30 min, washed, and allowed to recover for 4 h before fixation. For combination experiments, cells were treated with DMSO, HIT-4, Olaparib, or both, and fixed 4 h post-treatment.

Fixed cells were permeabilized with 0.2% Triton X-100 for 15 min, followed by blocking with 5% BSA in PBS for 1 h at room temperature. Cells were incubated overnight at 4 °C with anti-γ-H2AX primary antibody (#9718, 1:500 dilution). After washing with PBS, cells were incubated with Alexa Fluor 488-conjugated secondary antibody and DAPI (0.5 µg/mL) for 1 h at room temperature. Coverslips were mounted on glass slides using antifade mounting medium (Vector Laboratories), and images were acquired with a Leica TCS SP8 confocal microscope. γ-H2AX foci were quantified using Image-Pro Plus software across three independent experiments.

### Ethics

Gallbladder cancer specimens were collected from patients at Zhongshan Hospital of Fudan University (Shanghai, China), under a protocol approved by the Ethics Review Board of the hospital (B2024-541R). Written informed consent was obtained from all participants in accordance with regulatory standards prior to surgery. A total of 20 gallbladder cancer samples were collected and histopathologically verified at least two experienced pathologists according to the WHO classification guidelines [31].

All animal experiments were conducted in accordance with protocols approved by the Institutional Animal Care and Use Committee (IACUC) of Zhongshan Hospital of Fudan University. Female BALB/c nude mice (6–8 weeks old) were purchased from Vital River Laboratories (Beijing, China) and housed under specific pathogen-free conditions in individually ventilated cages with a 12-hour light/dark cycle and unrestricted access to food and water.

All experimental procedures adhered strictly to the relevant ethical guidelines and regulations.

### In vivo efficacy studies of NOZ-R and GBC-SD-R xenograft models

For subcutaneous tumor xenograft models, NOZ-R and GBC-SD-R cells (5 × 105 cells in 50 µL PBS mixed 1:1 with Matrigel) were injected subcutaneously into the right flank of BALB/c nude mice. Once tumor volumes reached approximately 100 mm³, mice were randomly assigned to four treatment groups (*n* = 5 per group): vehicle control (saline), HIT-4 (10 mg/kg, intravenous, three times per week), Olaparib (50 mg/kg, oral, three times per week), and the combination of HIT-4 and Olaparib. Tumor volumes and body weights were measured twice weekly. Tumor volume was calculated using the formula: volume = (length × width²)/2.

For survival analysis, an additional cohort of tumor-bearing mice (*n* = 5 per group) was monitored until tumors exceeded 1,000 mm³, at which point mice were euthanized as per humane endpoint criteria. Kaplan–Meier survival curves were plotted, and statistical significance was evaluated using the log-rank (Mantel–Cox) test. At the study endpoint, tumors were harvested for further histopathological and immunohistochemical analysis.

### Immunohistochemistry (IHC) and H-score analysis

Formalin-fixed, paraffin-embedded (FFPE) tumor tissues from 20 gallbladder cancer patients and from xenograft tumors derived from NOZ-R and GBC-SD-R models were processed for histological and immunohistochemical analyses. Hematoxylin and eosin (H&E) staining was performed to assess tissue morphology. For IHC, slides were dried at 62 °C for 1 h, followed by deparaffinization in xylene and rehydration through a graded ethanol series. Endogenous peroxidase activity was blocked with 3% hydrogen peroxide for 10 min. Antigen retrieval was performed in 10 mM citrate buffer (pH 6.0) by microwave heating for 20 min. After cooling and PBS washes, sections were blocked with 10% goat serum for 1 h at room temperature and then incubated overnight at 4 °C with primary antibodies against E2F8, RRM2, or cleaved caspase-3. After washing, sections were incubated with biotinylated secondary antibodies followed by streptavidin-HRP conjugates. Signal detection was achieved using diaminobenzidine (DAB) substrate solution, and nuclei were counterstained with hematoxylin. Slides were dehydrated in ascending ethanol, cleared in xylene, and mounted with coverslips.

IHC staining was evaluated using the semi-quantitative H-score method. Staining intensity was scored as 0 (negative), 1+ (weak), 2+ (moderate), or 3+ (strong), and the percentage of cells at each intensity was recorded. The H-score was calculated using the formula: H-score = 1 × (% of 1 + cells) + 2 × (% of 2 + cells) + 3 × (% of 3 + cells), resulting in a total score ranging from 0 to 300.

### Establishment of patient-derived organoids and drug treatment

Fresh gallbladder cancer (GBC) tissues were collected from six patients (three primary and three recurrent) with written informed consent and institutional ethical approval. Specimens were minced and digested in Collagenase IV (2.5 mg/mL) at 37 °C for 30–60 min with gentle agitation. Digestion was quenched with DMEM containing 10% fetal bovine serum. The suspension was filtered through a 100 μm strainer, centrifuged at 1000 rpm for 5 min, and washed twice with cold Advanced DMEM/F12 medium. Pelleted cells were resuspended in ice-cold Matrigel and seeded as domes in six-well suspension plates.

The organoid medium was Advanced DMEM/F12 supplemented with B27, N2, N-acetyl-L-cysteine (1.25 mM), Rspo-1 (250 ng/mL), Wnt3a (100 ng/mL), Noggin (100 ng/mL), EGF (50 ng/mL), FGF10 (100 ng/mL), IGF (100 ng/mL), HGF (25 ng/mL), gastrin (10 nM), Y-27,632 (10 µM), nicotinamide (10 mM), A83-01 (5 µM), forskolin (10 µM), Prostaglandin E2 (5 µg/mL), dexamethasone (3 nM), 1% penicillin/streptomycin, GlutaMAX and HEPES. For maintenance of GBC organoids, Wnt3a, Rspo-1, and Prostaglandin E2 were omitted.

For drug sensitivity assays, organoids were dissociated into small clusters or single cells using TrypLE Express, resuspended in complete medium containing 5% Matrigel, and seeded into 96-well ultra-low-attachment plates (100 µL per well). After overnight recovery, cells were treated with DMSO, HIT-4, Olaparib, or their combination at the indicated concentrations. Cell viability was measured 5 days after treatment using the CellTiter-Glo 3D Cell Viability Assay according to the manufacturer’s protocol.

### Statistics

All experiments were independently replicated at least three times unless otherwise indicated. Statistical evaluations were conducted using GraphPad Prism (version 10). Data for in vitro studies are presented as mean ± standard deviation (SD), and results from in vivo studies are depicted as mean ± standard error of the mean (SEM) unless otherwise stated. Differences between two groups were examined using Student’s t-tests (two-sided, either paired or unpaired). For analyses involving multiple groups (three or more), one-way or two-way ANOVA tests were applied. Pearson’s correlation coefficient (r) was employed to evaluate correlations, and associated p-values were computed using Prism software. IC_50_ values were calculated by nonlinear regression analysis using log-transformed datasets. Kaplan-Meier survival analysis was employed to assess survival distributions, with statistical significance determined via log-rank tests.

## Results

### CRISPR KO screening reveals genes regulating PARP inhibitor sensitivity in resistant GBC cells

**Fig. 1:**
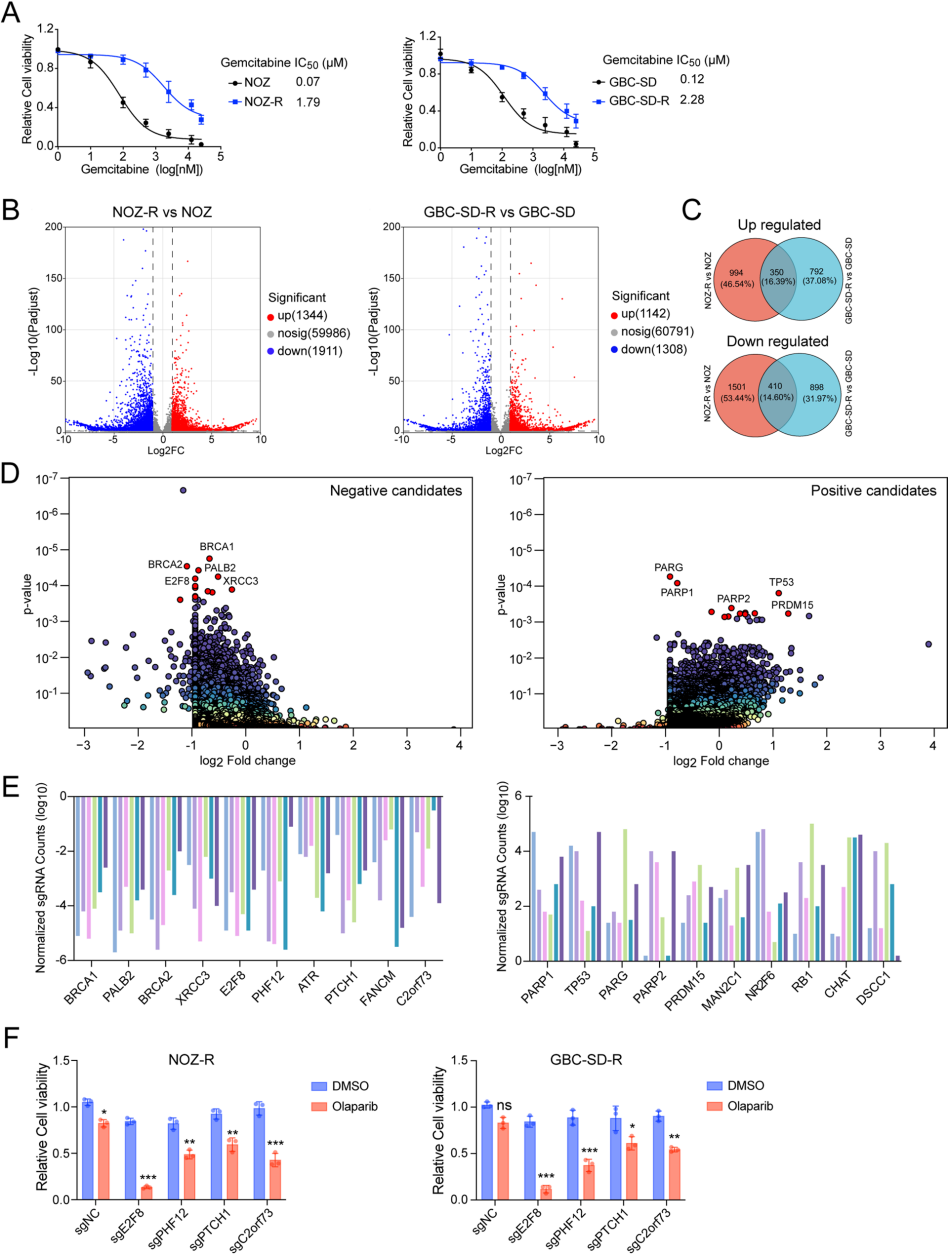
Generation of gemcitabine-resistant GBC cell lines and genome-wide CRISPR screen for therapeutic targets (**A**) Dose-response curves and IC_50_ values of gemcitabine in parental (NOZ and GBC-SD) and gemcitabine-resistant (NOZ-R and GBC-SD-R) cells, assessed by CellTiter-Glo viability assays after 5 days treatment. Data are presented as mean ± SD from three independent experiments (**B**) Volcano plots of differentially expressed genes (DEGs): NOZ-R versus NOZ (left) and GBC-SD-R versus GBC-SD (right). Significantly upregulated (red) and downregulated (blue) genes are defined by log_2_ fold-change > 1 or < −1 and adjusted *p* < 0.05 (**C**) Venn diagrams illustrating the overlap of significantly upregulated (top) and downregulated (bottom) DEGs in NOZ-R and GBC-SD-R cells (**D**) Genome-wide CRISPR-Cas9 loss-of-function screen in NOZ-R cells under Olaparib versus DMSO treatment, highlighting top candidate genes with significant depletion (negative selection, left) or enrichment (positive selection, right) based on fold-change and *p*-value (**E**) Normalized sgRNA counts for the top 10 candidate genes identified from the CRISPR screen, with six sgRNAs per gene (**F**) Validation of selected screen hits (E2F8, PHF12, PTCH1 and C2orf73) by CRISPR-mediated knockout in NOZ-R and GBC-SD-R cells, followed by treatment with Olaparib (1 µM) or DMSO for 5 days. Cell viability was measured using CellTiter-Glo. Data represent mean ± SD from three independent experiments. Statistical significance was determined using two-tailed Student’s t-test (*p < 0.05, **p < 0.01, ***p < 0.001; ns: not significant)

To elucidate the mechanisms underlying gemcitabine resistance in gallbladder cancer (GBC), we established two gemcitabine-resistant cell lines, NOZ-R and GBC-SD-R, derived from their parental counterparts, NOZ and GBC-SD, respectively. Cell viability assays confirmed that NOZ-R and GBC-SD-R cells exhibited significantly elevated IC_50_ values for gemcitabine (1.79 µM and 2.28 µM, respectively) compared to their parental lines (0.07 µM for NOZ and 0.12 µM for GBC-SD). Accordingly, NOZ-R and GBC-SD-R cells exhibited approximately 25- and 19-fold increases in gemcitabine resistance compared with their parental lines, validating the establishment of resistance (Fig. 1A). Transcriptomic profiling via RNA sequencing revealed widespread gene expression alterations between resistant and parental cells. In NOZ-R versus NOZ, 1,344 genes were significantly upregulated and 1,911 downregulated; similarly, in GBC-SD-R versus GBC-SD, 1,142 genes were upregulated and 1,308 downregulated (Fig. 1B and Supplementary Tables 1–2). Venn diagrams-based intersection analysis identified 350 commonly upregulated and 410 commonly downregulated genes shared by both resistant cell lines (Fig. 1C and Supplementary Table 3). KEGG pathway enrichment analysis of differentially expressed genes (DEGs) highlighted a strong enrichment in DNA repair-related pathways, including cell cycle regulation, mismatch repair, and homologous recombination, across both individual and overlapping DEG sets (Supplementary Fig. 1A-C).

To identify genetic vulnerabilities under PARP inhibition, we conducted a genome-wide CRISPR/Cas9 knockdown screen in NOZ-R cells treated with either Olaparib or DMSO. Comparative analysis revealed a panel of candidate genes whose loss significantly altered cellular sensitivity to Olaparib (Supplementary Table 4). Among negatively selected hits, well-established DNA repair genes such as *BRCA1/2*, *PALB2*, and *ATR* were identified, while *PARP1* and *PARP2* emerged as positively selected candidates (Fig. 1D, E), confirming the robustness and reliability of the screening system. KEGG enrichment analysis of these candidate genes further supported the involvement of DNA repair pathways in modulating PARP inhibitors response (Supplementary Fig. 1D). From the top-ranked negatively selected genes, we examined four underexplored candidates - *E2F8*, *PHF12*, *PTCH1*, and *C2orf73* – for further functional validation. CRISPR-mediated knockout of these genes significantly enhanced Olaparib sensitivity in both NOZ-R and GBC-SD-R cells, as measured by cell viability assays. Among them, *E2F8* knockout led to the most pronounced reduction in cell viability—approximately 80% in both cell lines—under combined treatment conditions, indicating its pivotal role in modulating PARP inhibitor efficacy in gemcitabine-resistant GBC cells (Fig. 1F). Collectively, these findings provide a comprehensive map of genetic factors influencing PARP inhibitor sensitivity in gemcitabine-resistant GBC and identify *E2F8* as a promising therapeutic target.

### E2F8 depletion disrupts DNA repair and enhances PARP inhibitor sensitivity

**Fig. 2:**
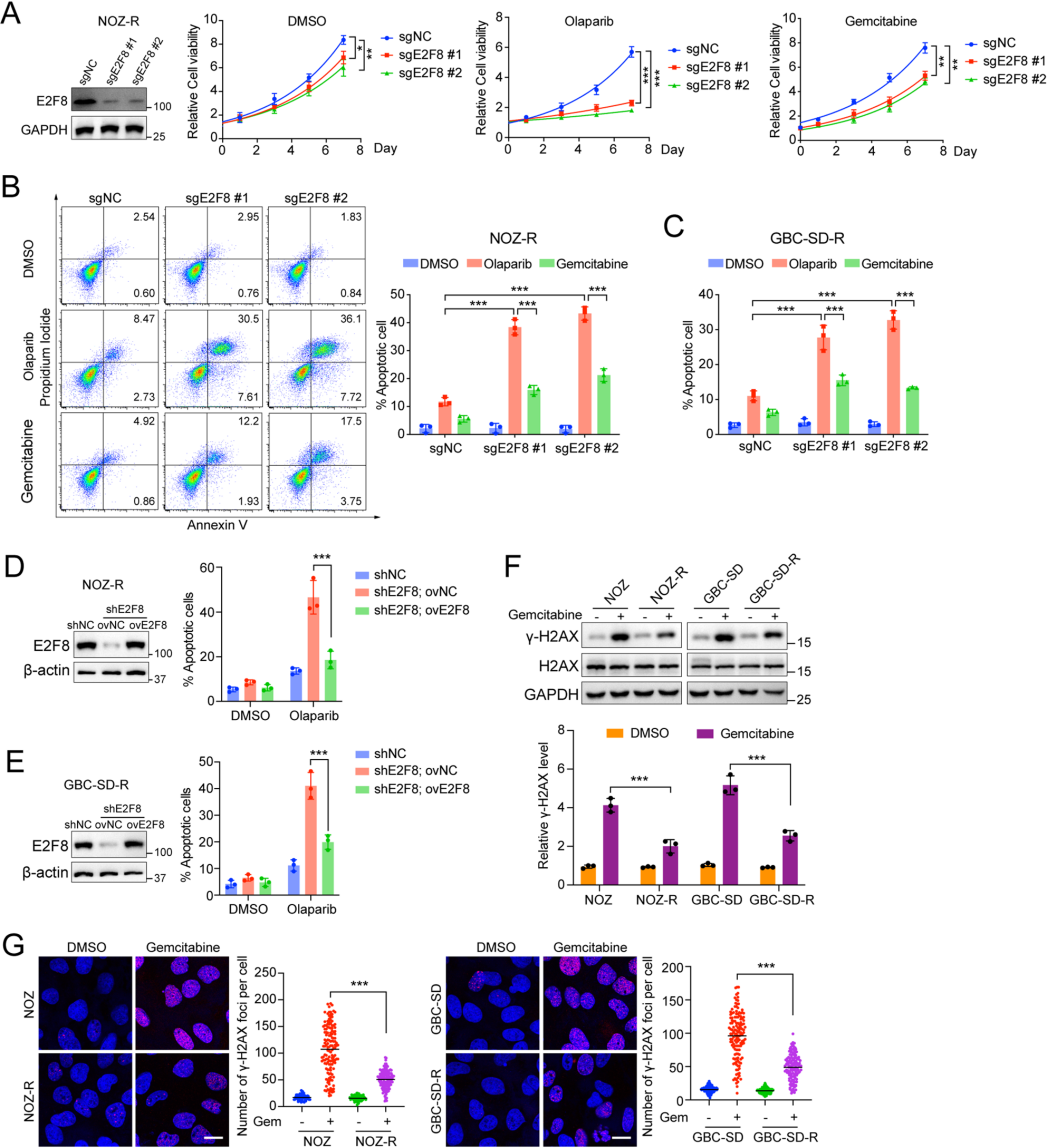
E2F8 deficiency sensitizes gemcitabine-resistant gallbladder cancer cells to PARP inhibition. (**A**) Knockdown of E2F8 enhances sensitivity to Olaparib and gemcitabine in NOZ-R cells. Cells were transduced with control sgRNA (sgNC) or two independent sgRNAs targeting E2F8 (sgE2F8 #1 and sgE2F8 #2), followed by treatment with DMSO, Olaparib (1 µM), or gemcitabine (0.1 µM). Cell viability was measured at the indicated time points using the CellTiter-Glo assay. Data are shown as mean ± SD from three independent experiments (two-tailed t-test; *p < 0.05, **p < 0.01, ***p < 0.001) (**B**) Apoptosis analysis of NOZ-R cells transduced with sgNC or sgE2F8 (#1 and #2) and treated with DMSO, Olaparib (1 µM), or gemcitabine (0.1 µM) for three days. Apoptotic cells were detected by Annexin V/PI staining followed by flow cytometry. Data are presented as mean ± SD from three independent experiments (t-test; ***p < 0.001) (**C**) Quantification of apoptotic cells in GBC-SD-R cells transduced with sgNC or sgE2F8 (#1 and #2) and treated with DMSO, Olaparib (1 µM), or gemcitabine (0.1 µM) for three days, as determined by Annexin V/PI staining and flow cytometry (**D**-**E**) Apoptosis assays in NOZ-R (**D**) and GBC-SD-R (**E**) cells stably expressing control (shNC) or E2F8-targeting shRNA (shE2F8), with or without E2F8 overexpression, followed by treatment with DMSO or Olaparib (1 µM). Apoptotic cells were quantified by flow cytometry after Annexin V/PI staining. E2F8 expression levels were confirmed by western blotting (**F**) Western blot analysis of γ-H2AX levels in NOZ, NOZ-R, GBC-SD, and GBC-SD-R cells pretreated with gemcitabine (0.5 µM) for 30 min and harvested 4 h later. Quantitative of γ-H2AX levels was performed using ImageJ software and normalized to total H2AX (**G**) Immunofluorescence detection and quantification of γ-H2AX foci per nucleus in the same panel of cell lines treated as in (F). Foci were quantified using Image-Pro Plus software. Scatter dot plots represent mean ± SD (*n* = 3 independent experiments, unpaired t-test; ***p < 0.001)

Given that E2F8 has been reported to correlate with poor prognosis and chemoresistance in various cancers [18, 20, 22], we next explored its role in modulating the sensitivity of GBC cells to PARP inhibition. To this end, we established E2F8-deficient gemcitabine-resistant GBC cell lines (NOZ-R and GBC-SD-R) by transducing with two independent sgRNAs targeting E2F8. Knockdown of E2F8 significantly enhanced sensitivity to the PARP inhibitor Olaparib, as evidenced by a marked reduction in cell viability compared to controls cells (Fig. 2A and Supplementary Fig. 2A). Similarly, E2F8 knockdown also increased the sensitivity of these cells to gemcitabine, further supporting its role in mediating drug resistance. Consistently, Annexin V/PI staining revealed a substantial increase in apoptotic cell populations upon Olaparib or gemcitabine treatment in E2F8-deficient NOZ-R and GBC-SD-R cells relative to controls, although the apoptotic effect of gemcitabine remained weaker than that of Olaparib (Fig. 2B-C and Supplementary Fig. 2B). Notably, this enhanced sensitivity to Olaparib was not observed in parental NOZ and GBC-SD cells, which express low endogenous E2F8 levels compared to their resistant counterparts (Supplementary Fig. 2C). To further validate the role of E2F8 in PARP inhibitor sensitivity, we generated stable GBC cell lines with E2F8 knockdown (shE2F8) and reconstituted E2F8 expression (ovE2F8) in the same background. As expected, E2F8 knockdown led to increased apoptosis, which was effectively reversed upon E2F8 re-expression (Fig. 2D-E), confirming the functional importance of E2F8 in mediating resistance to PARP inhibition.

Given the known involvement of DNA damage response (DDR) in both gemcitabine resistance and PARP inhibitor efficacy [32, 33], we next assessed DDR status by evaluating γ-H2AX levels. Western blot analysis revealed that parental NOZ and GBC-SD cells exhibited significantly higher γ-H2AX levels upon gemcitabine treatment compared to resistant NOZ-R and GBC-SD-R cells, suggesting that the resistant cells possess enhanced DNA repair capacity (Fig. 2F). Immunofluorescence further confirmed a marked reduction in γ-H2X foci in resistant cells relative to parental lines (Fig. 2G). Importantly, E2F8 knockdown in resistant cells restored γ-H2AX foci formation following gemcitabine treatment, whereas E2F8 re-expression abrogated this effect (Supplementary Fig. 2D), highlighting a critical role of E2F8 in facilitating DNA repair processes in resistant GBC cells. Together, these findings demonstrate that E2F8 promotes resistance to gemcitabine by enhancing DNA repair, and its depletion sensitizes resistant GBC cells to PARP inhibitor through impaired DNA damage repair.

### E2F8 regulates PARP inhibitor sensitivity via RRM2

**Fig. 3:**
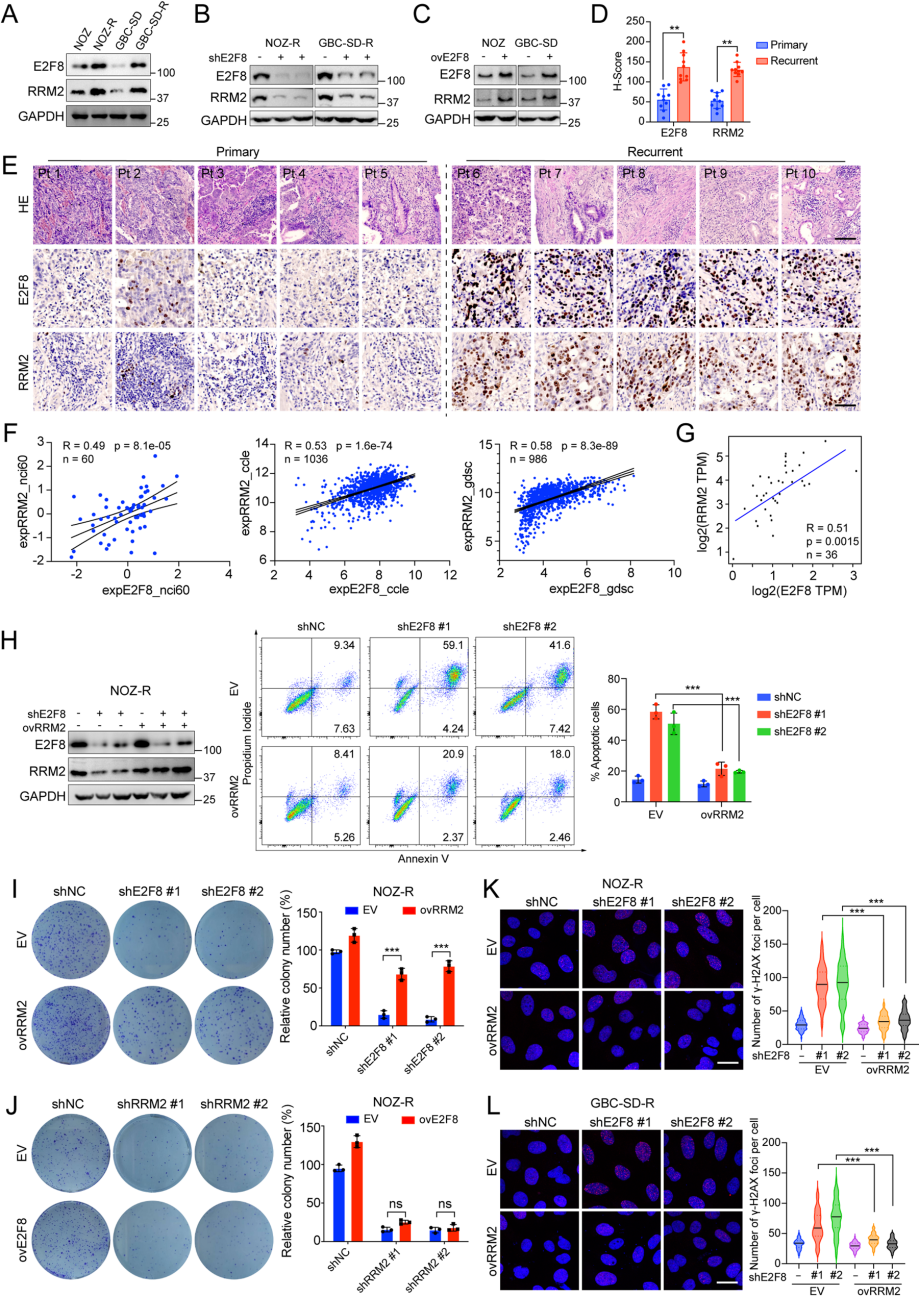
The E2F8-RRM2 axis promotes DNA repair and confers drug resistance in GBC cells (**A**) Western blot analysis of E2F8 and RRM2 protein expression in parental (NOZ, GBC-SD) and gemcitabine-resistant (NOZ-R, and GBC-SD-R) cells. GAPDH was used as a loading control (**B**) Knockdown of E2F8 using two independent shRNAs (shE2F8 #1 and #2) reduces RRM2 protein levels in NOZ-R and GBC-SD-R cells compared to non-targeting control (shNC) (**C**) Ectopic expression of E2F8 increases RRM2 protein levels in NOZ and GBC-SD cells, as shown by Western blotting (**D**) Quantification of E2F8 and RRM2 IHC staining in primary and recurrent gallbladder cancer tissues. H-scores were calculated from 10 patients per group, with recurrent cases having received gemcitabine and cisplatin treatment. Scatter dot plots represent mean ± SD (unpaired t-test; **p < 0.01) (**E**) Representative H&E (hematoxylin and eosin) and IHC staining images of E2F8 and RRM2 in tumor tissues from 10 gallbladder cancer patients. Samples from patients 1–5 (Pt1-5) represent primary tumors, whereas those from patients 6–10 (Pt6-10) represent recurrent tumors. Scale bars: H&E, 100 μm; IHC, 50 μm (**F**) Cross-platform correlation analyses reveal a consistent positive association between E2F8 and RRM2 expression across NCI60, CCLE, and GDSC cell line datasets (**G**) E2F8 and RRM2 mRNA levels are positively correlated in the TCGA cholangiocarcinoma cohort (**H**) E2F8 knockdown sensitizes NOZ-R cells to Olaparib (1 µM, 3 days), an effect that is reversed by RRM2 overexpression. E2F8 and RRM2 protein levels were confirmed by Western blotting. Apoptotic cells were quantified by Annexin V/PI staining and flow cytometry. Data are presented as mean ± SD from three independent experiments (t-test; ***p < 0.001) (**I**) Colony formation assays show that overexpression of RRM2 rescues the impaired proliferative capacity of E2F8-knockdown NOZ-R cells (**J**) E2F8 overexpression fails to rescue the proliferation defect induced by RRM2 knockdown in NOZ-R cells (K-L) Representative images and quantification of γ-H2AX foci in NOZ-R (**K**) and GBC-SD-R (**L**) cells. Cells with stable E2F8 knockdown (shE2F8 #1 and #2) or shNC, with or without RRM2 overexpression, were treated with Olaparib (1 µM) for 4 h prior to fixation and staining. Data are shown as mean ± SD (*n* = 3 independent experiments, unpaired t-test; ***p < 0.001)

To investigate the mechanisms by which E2F8 contributes to DNA repair and gemcitabine resistance in GBC cells, we first analyzed RNA-seq data and observed that RRM2 - a known downstream effector of E2F8 involved in DNA replication and repair [24, 34] - was also upregulated in resistant GBC cell lines. Consistently, Western blot analysis demonstrated a positive correlation between E2F8 and RRM2 protein expression in NOZ and GBC-SD parental cells and their resistant derivatives, NOZ-R and GBC-SD-R, where both proteins were notably elevated in the resistant lines (Fig. 3A). Knockdown of E2F8 in NOZ-R and GBC-SD-R cells led to a marked reduction in RRM2 expression (Fig. 3B), while ectopic expression of E2F8 in parental cells resulted in a corresponding increase in RRM2 levels (Fig. 3C), confirming the regulatory relationship between E2F8 and RRM2. We further validated this correlation in vivo by performing immunohistochemical (IHC) analyses on tumor specimens from 20 GBC patients, including 10 primary and 10 recurrent cases (the latter of which had received gemcitabine and cisplatin treatment) **(Supplementary Table 5)**. Quantification of IHC H-scores indicated that both E2F8 and RRM2 were significantly upregulated in recurrent compared with primary gallbladder cancers (Fig. 3D-E). This association was corroborated by analyses of public datasets, including NCI60, CCLE, GDSC, and the TCGA cholangiocarcinoma cohort [35, 36], reinforcing the clinical and biological relevance of the E2F8-RRM2 axis (Fig. 3F-G).

To determine whether RRM2 mediates the functional effect of E2F8, we restored RRM2 expression in E2F8-silenced NOZ-R and GBC-SD-R cells. The pro-apoptotic effect induced by E2F8 knockdown in response to PARP inhibition was effectively rescued by RRM2 overexpression (Fig. 3H and Supplementary Fig. 3A). Similarly, colony formation assays demonstrated that the impaired proliferative capacity caused by E2F8 depletion was reversed by RRM2 re-expression (Fig. 3I and Supplementary Fig. 3B). In contrast, overexpress of E2F8 failed to rescue proliferation in RRM2-silenced cells (Fig. 3J and Supplementary Fig. 3C-D), suggesting that RRM2 functions downstream of E2F8. Further supporting this model, γ-H2AX foci formation assays revealed that E2F8 knockdown increased DNA damage, which was mitigated by RRM2 overexpression (Fig. 3K-L). Conversely, RRM2 knockdown elevated γ-H2AX levels, and this phenotype could not be rescued by E2F8 overexpression (Supplementary Fig. 3E). These findings collectively demonstrate that RRM2 is a critical downstream effector of E2F8 in mediating DNA repair, chemoresistance, and cell survival in GBC. This highlights the therapeutic potential of targeting the E2F8-RRM2 axis to overcome drug resistance.

### Identification and characterization of HIT-4 as a potent inhibitor disrupting the E2F8-DNA interaction

**Fig. 4:**
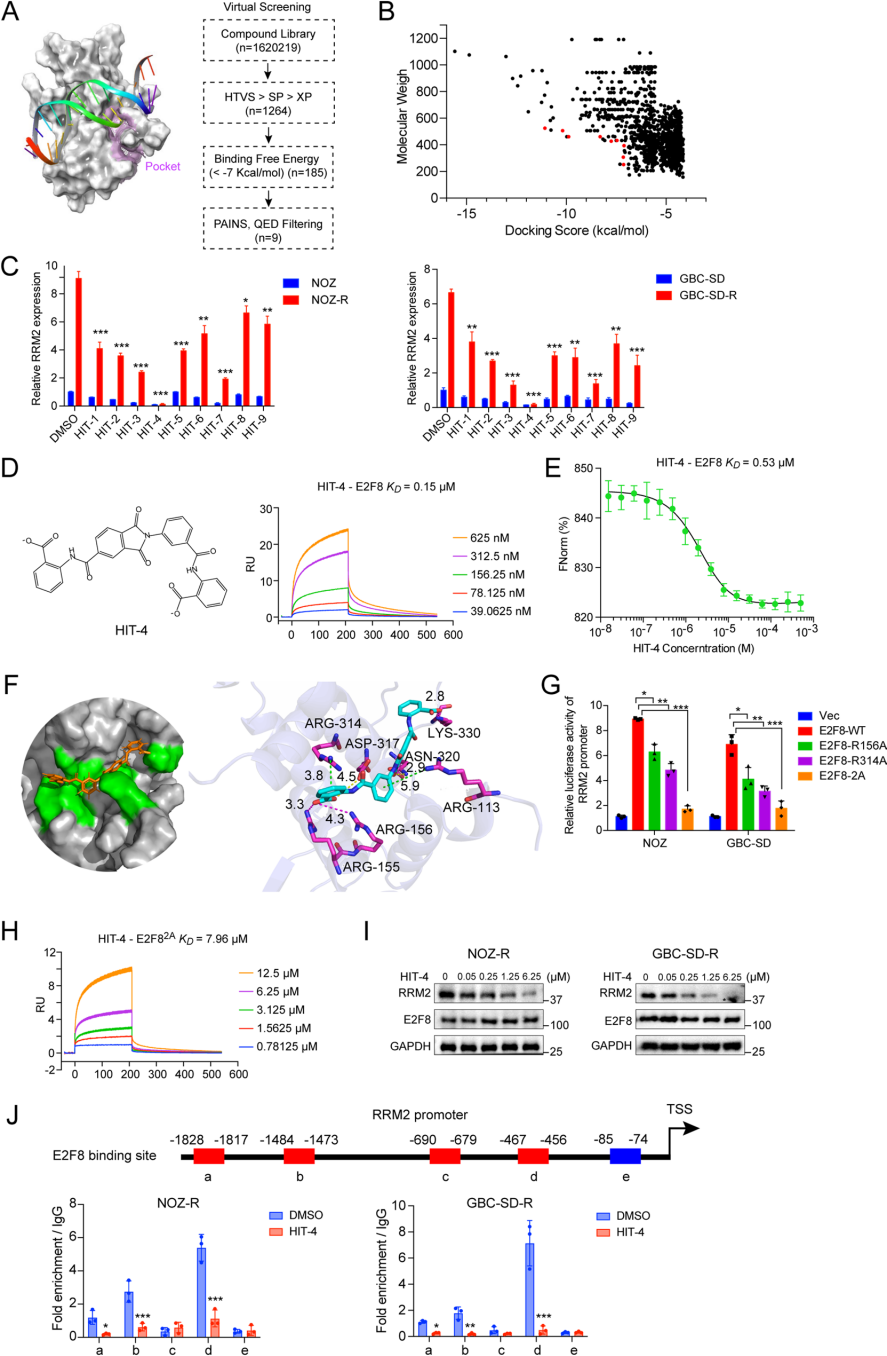
Identification of HIT-4 as an inhibitor of E2F8-DNA interaction through high-throughput virtual screening. (**A**) Schematic illustration of the E2F8 DNA-binding pocket (purple, left), and the virtual screening workflow (right) using Schrödinger software. A compound library of over 1.62 million small molecules was subjected to high-throughput virtual screening (HTVS), followed by standard precision (SP) and extra precision (XP) docking. Compounds were further filtered based on binding free energy (< −7 kcal/mol), pan-assay interference structures (PAINS), and quantitative estimation of drug-likeness (QED), yielding 9 candidate compounds (**B**) Scatter plot of molecular weight versus docking scores for 1,264 compounds after HTVS-SP-XP screening. The top 9 hits are highlighted in red (**C**) RT-PCR analysis of RRM2 mRNA levels in NOZ/NOZ-R (left) and GBC-SD/GBC-SD-R (right) cells following treatment with each of the top 9 candidate compounds (HIT-1 to HIT-9; 10 µM for 6 h). DMSO was used as the control. Data are presented as mean ± SD from three independent experiments (t-test; *p < 0.05, **p < 0.01, ***p < 0.001) (**D**) Chemical structure of HIT-4 (left) and surface plasmon resonance (SPR) analysis of its binding affinity to E2F8 (right). E2F8 was immobilized on a CM5 sensor chip, and kinetic constants were calculated using Biacore 8 K evaluation software with a 1:1 steady-state affinity model. RU, resonance units (**E**) Dose-response curve of HIT-4 binding to E2F8 measured by MicroScale Thermophoresis (MST). Experiments were conducted in triplicate using 80% excitation and 40% MST power. Binding constants were calculated using MO. Affinity Analysis software. FNorm, normalized fluorescence (**F**) Molecular docking model of HIT-4 binding to the E2F8 DNA-binding pocket (left), with a detailed interaction view (right) showing key interacting residues and molecular distances (**G**) Luciferase reporter assays in NOZ and GBC-SD cells transfected with wild-type E2F8, single-point mutants (R156A, R314A), or a double mutant (R156A/R314A), along with an RRM2 promoter-driven luciferase construct. Luminescence was measured 6 h post-transfection. Data are shown as mean ± SD from three independent experiments (two-tailed t-test; *p < 0.05, **p < 0.01, ***p < 0.001) (**H**) SPR analysis of HIT-4 binding affinity to E2F8 mutant (R156A and R314A) (**I**) Western blot analysis of RRM2 protein levels in NOZ-R and GBC-SD-R cells after treatment with increasing concentrations of HIT-4 (**J**) Schematic representation of predicted E2F8 binding sites (red) and a negative control region (blue) within the RRM2 promoter based on JASPAR prediction (top). ChIP-qPCR was performed to assess E2F8 enrichment at these sites in NOZ-R and GBC-SD-R cells treated with HIT-4 (1 µM) or DMSO for 6 h. Data are presented as mean ± SD from three independent experiments (t-test; *p < 0.05, **p < 0.01, ***p < 0.001)

Given the therapeutic relevance of E2F8 in gemcitabine-resistant GBCs and its well-defined DNA-binding interface revealed by co-crystal structure [29], we pursued a structure-based strategy to identify small-molecule inhibitors that block E2F8-DNA interactions. Using Schrödinger software, a high-throughput virtual screening (HTVS) campaign was conducted starting from over 1.62 million small molecules. A sequentially triage involving HTVS, standard precision (SP), and extra precision (XP) docking yielded 1,264 candidate compounds (Supplementary Table 6). These were further refined based on binding free energy, PAINS filtering, and quantitative estimate of drug-likeness (QED), ultimately resulting in 9 promising candidates (Fig. 4A-B and Supplementary Fig. 4A).

We next assessed the functional impact of these candidates on RRM2, a direct downstream target of E2F8. Compounds HIT-3, HIT-4, and HIT-7 exhibited the strongest inhibition of RRM2 expression across both parental (NOZ, GBC-SD) and gemcitabine-resistant (NOZ-R, GBC-SD-R) GBC cell lines (Fig. 4C**)**. To confirm direct engagement with E2F8, we performed surface plasmon resonance (SPR) binding assays. HIT-4 showed the highest affinity (*K*_*D*_ = 0.15 µM), while HIT-3 and HIT-7 exhibited moderate binding (*K*_*D*_ = 10.5 µM and 6.24 µM, respectively) (Fig. 4D and Supplementary Fig. 4B). Microscale thermophoresis (MST) assays further validated the strong interaction of HIT-4 with E2F8, yielding a *K*_*D*_ of 0.53 µM (Fig. 4E).

As co-crystallization attempts of the E2F8-HIT-4 complex were unsuccessful, we employed molecular docking to predict the binding mode. Docking analysis identified R156 and R314 within the DNA-binding domain of E2F8 as key residues mediating HIT-4 interaction (Fig. 4F and Supplementary Fig. 4C). To functionally validate this model, site-directed mutagenesis of R156 and R314 was performed. Luciferase reporter assays demonstrated impaired activation of the *RRM2* promoter by the E2F8 mutants, supporting the functional relevance of these residues (Fig. 4G). SPR analysis further confirmed that the R156A/R314A mutant displayed markedly reduced binding affinity to HIT-4 (*K*_*D*_ = 7.96 µM) (Fig. 4H). To determine whether HIT-4 functionally disrupts E2F8 transcriptional activity, we treated NOZ-R and GBC-SD-R cells with increasing doses of HIT-4. Western blot and RT-PCR analyses revealed a dose-dependent suppression of RRM2 expression at both the protein and mRNA levels, with no effect on E2F8 protein levels (Fig. 4I and Supplementary Fig. 4D). Finally, chromatin immunoprecipitation followed by qPCR (ChIP-qPCR) revealed strong E2F8 enrichment at region “d” of the *RRM2* promoter, which was significantly reduced upon HIT-4 treatment (Fig. 4J), confirming that HIT-4 disrupts the E2F8-DNA interaction at the endogenous chromatin level. Collectively, these findings strongly identify HIT-4 as a potent and selective inhibitor of E2F8, capable of blocking its DNA-binding activity and downstream transcriptional regulation of RRM2, offering a promising therapeutic strategy for overcoming chemoresistance in GBC.

### HIT-4 enhances Olaparib sensitivity in E2F8-overexpressing resistant GBC cells

**Fig. 5:**
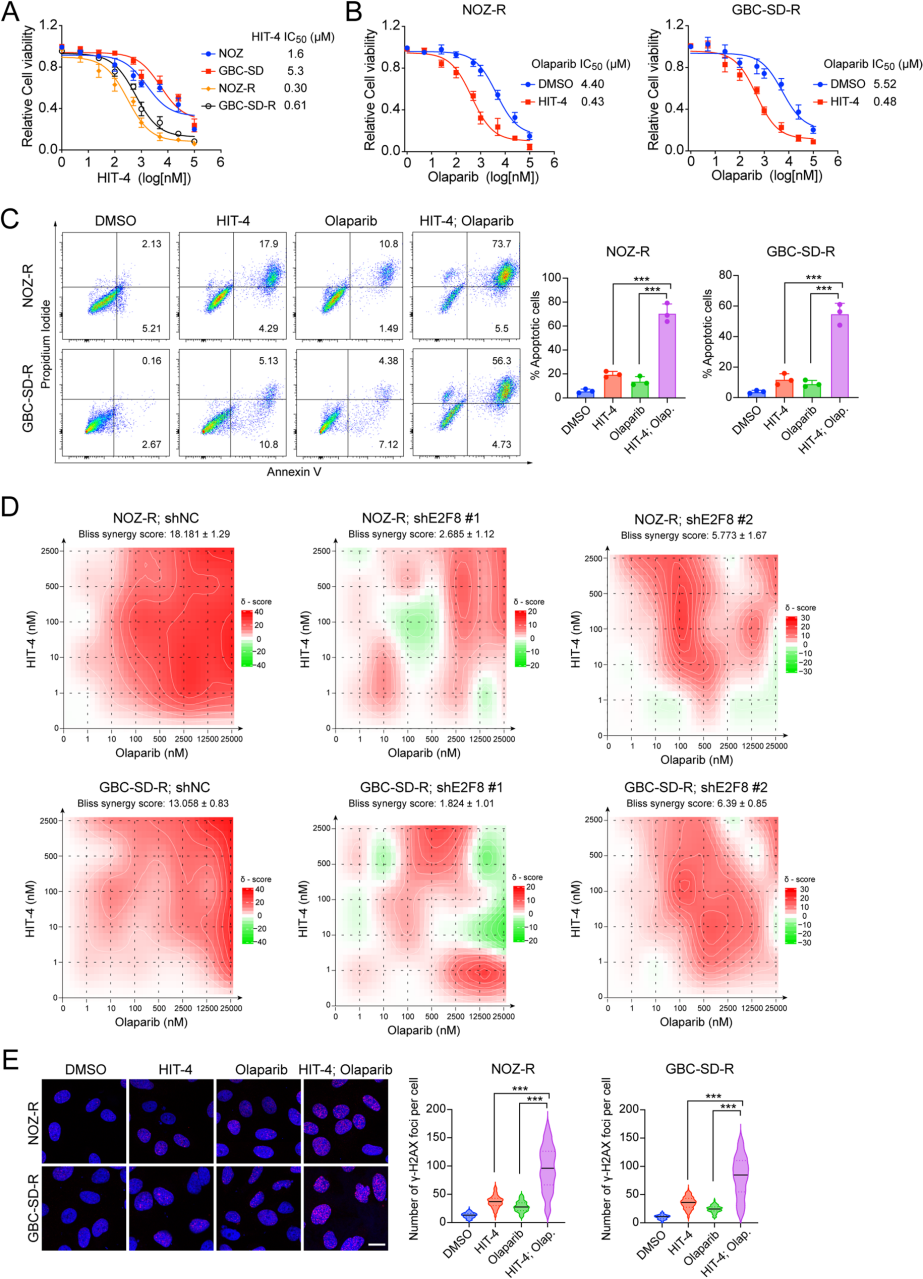
HIT-4 sensitizes E2F8-overexpressing, drug-resistant tumor cells to PARP inhibition. (**A**) Dose-response curves of E2F8-overexpressing NOZ-R and GBC-SD-R cells compared to their parental NOZ and GBC-SD cells following HIT-4 treatment for 5 days. Cells viability was assessed using the CellTiter-Glo assay, and IC_50_ values were calculated using GraphPad Prism 10. Data are presented as mean ± SD from three independent experiments (**B**) Dose-response curves of NOZ-R and GBC-SD-R cells treated with Olaparib in the presence of either DMSO or HIT-4 (1 µM). Cell viability was measured by CellTiter-Glo assay (**C**) Quantification of apoptotic cells in NOZ-R and GBC-SD-R cells treated with HIT-4 (1 µM), Olaparib (1 µM), or the combination for 3 days, followed by Annexin V/PI staining and flow cytometry. Data are shown as mean ± SD (*n* = 3 independent experiments; two-way ANOVA; ***p < 0.001) (**D**) Bliss synergy maps illustrating the combinatorial effect of HIT-4 and Olaparib in shNC or shE2F8 (#1 and #2) NOZ-R and GBC-SD-R cells. Cells were treated for 96 h in biological triplicates. Bliss synergy scores greater than 10 indicate strong synergy (**E**) Representative immunofluorescence images and quantification of γ-H2AX foci in NOZ-R and GBC-SD-R cells treated with HIT-4 (1 µM), Olaparib (1 µM), or the combination for 4 h. Data are shown as mean ± SD (*n* = 3 independent experiments, unpaired t-test; ***p < 0.001). Scale bar: 10 μm

To determine the therapeutic efficacy of HIT-4 in GBC cells exhibiting gemcitabine resistance and elevated E2F8 expression, we evaluated its anti-proliferative effects. Dose-response analyses revealed that HIT-4 significantly reduced IC_50_ values in gemcitabine-resistant NOZ-R and GBC-SD-R cell lines compared to their parental counterparts, NOZ and GBC-SD, indicating heightened sensitivity to HIT-4 in cells with E2F8 overexpression (Fig. 5A). To assess the broader applicability of HIT-4, we examined its effect on a panel of cancer cell lines, including SW1353, CACO-2, MDA-MB-231, UACC-893, D341 and Daoy. As shown in Supplementary Fig. 5A, HIT-4 treatment induced notable growth inhibition, with IC_50_ values ranging from 0.22 µM to 4.39 µM. Sensitivity to HIT-4 correlated with endogenous E2F8 protein levels, as confirmed by Western blot analysis alongside RRM2 expression profiling. Differential responses across the tested lines were consistent with their respective E2F8 expression levels. Next, we investigated the combinatorial effects of HIT-4 and the PARP inhibitor. Co-treatment with HIT-4 markedly lowered the IC_50_ of Olaparib in NOZ-R (from 4.4 µM to 0.43 µM) and GBC-SD-R (from 5.52 µM to 0.48 µM) cells, indicating a potent synergistic effect (Fig. 5B). In contrast, this effect was not observed in parental NOZ and GBC-SD cells (Supplementary Fig. 5B). Flow cytometry using Annexin V/PI staining further revealed significantly increased apoptosis in resistant cells receiving combination treatment compared to either agent alone (Fig. 5C).

To quantitatively confirm synergy, Bliss independence scores were calculated using SynergyFinder [37]. In NOZ-R and GBC-SD-R cells, HIT-4 and Olaparib exhibited robust synergy, with Bliss scores exceeding 10 (Fig. 5D). Notably, E2F8 knockdown abrogated this synergy, underscoring E2F8’s critical role in mediating the combinatorial effect. In parental cells, Bliss synergy remained low, indicating minimal or no synergistic interactions (Supplementary Fig. 5C). In addition, HIT-4 exhibited only a modest synergistic effect with gemcitabine, in contrast to the pronounced synergy observed with Olaparib (Supplementary Fig. 5D). To further confirm the DNA damage induced by combined therapy, immunofluorescence staining of γ-H2AX was performed. A significant increase in γ-H2AX foci formation was observed in NOZ-R and GBC-SD-R cells following combined treatment with HIT-4 and Olaparib, supporting enhanced DNA damage in these conditions (Fig. 5E). Consistently, colony formation assays demonstrated significant suppression of clonogenic potential in resistant cells under combination treatment, while parental and low E2F8-expressing lines such as SW1353 and Daoy exhibited negligible responses (Supplementary Fig. 5E-F). To determine whether this synergy requires an HR-defective background, we examined *BRCA2*-mutant Capan-1 (pancreatic) and *BRCA1*-mutant UWB1.289 (ovarian) cell lines [38, 39]. As expected, these HR-deficient cells were already highly sensitive to Olaparib alone, and no further reduction in IC_50_ was observed upon HIT-4 co-treatment (Supplementary Fig. 5G). Taken together, these results indicate that HIT-4 selectively targets E2F8-overexpressing, gemcitabine-resistant GBC cells and synergistically potentiates the antitumor activity of Olaparib. This synergy appears to be independent of pre-existing HR-defects and is instead mediated by HIT-4 induced suppression of RRM2-driven DNA repair. Collectively, this combination represents a promising therapeutic strategy for resistant malignancies characterized by high E2F8 expression.

### HIT-4 synergizes with Olaparib to suppress tumor growth via E2F8-mediated RRM2 Inhibition in vivo

**Fig. 6:**
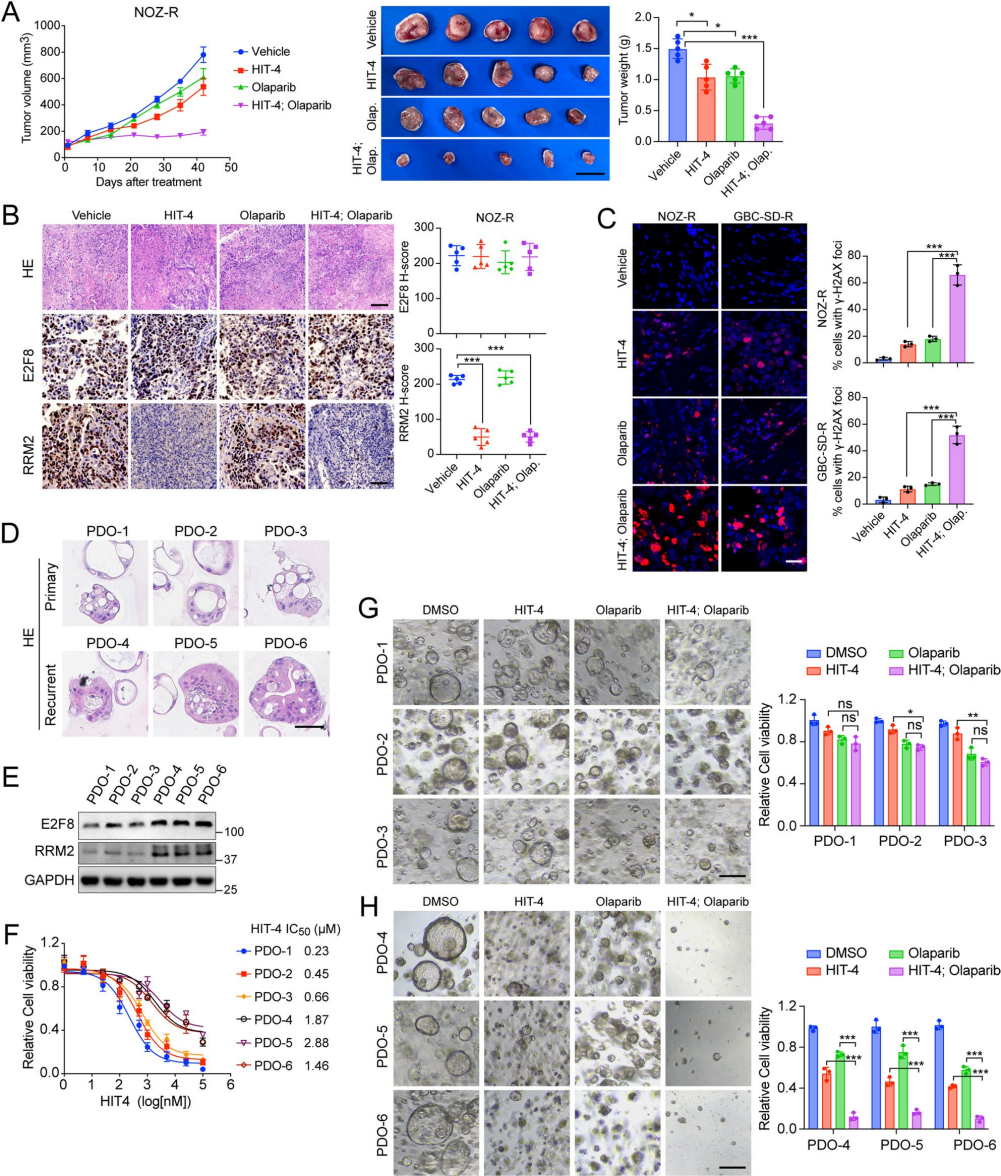
HIT-4 sensitizes gemcitabine-resistant GBC to PARP inhibition through RRM2 suppression (**A**) Combination treatment with HIT-4 and Olaparib significantly reduces tumor growth in NOZ-R subcutaneous xenograft models. A total 5 × 10^5^ cells were injected into the right flank of BALB/c nude mice, followed by random assignment to receive vehicle, HIT-4 (10 mg/kg), Olaparib (50 mg/kg), or the combination. Tumor volumes were measured weekly, and tumors were harvested and weighed after 42 days of treatment. Data are represented as mean ± SD (two-way ANOVA; * *p* < 0.05, *** *p* < 0.001). Scale bar: 1 cm (**B**) Representative H&E and IHC staining of xenograft tumors tissues from NOZ-R models corresponding to (A). H-Scores for E2F8 and RRM2 expression were quantified from tumors of five individual mice per group. Data are represented as mean ± SD (two-way ANOVA; *** *p* < 0.001). Scale bar, 100 μm (**C**) Immunofluorescence staining of γ-H2AX in NOZ-R and GBC-SD-R xenograft tumors. Cells exhibiting more than 20 γ-H2AX foci were counted, and the percentage of γ-H2AX-positive cells was quantified. Statistical analysis was performed using two-way ANOVA. Scale bar, 20 μm (**D**) Representative H&&E staining in PDOs derived from primary (PDO1-3) and recurrent (PDO4-6) tumors. Scale bar: 50 μm (**E**) Western blot analysis of E2F8 and RRM2 expression in PDOs. GAPDH served as a loading control (**F**) Dose-response curves of PDOs treated with HIT-4 for 5 days. Cell viability was assessed using the CellTiter-Glo assay, and IC_50_ values were calculated with GraphPad Prism 10. Data are presented as mean ± SD from three independent experiments (**G**-**H**) Representative images and quantification of cell viability in primary and recurrent PDOs treated with HIT-4, Olaparib, or their combination for 5 days. Data are shown as mean ± SD (*n* = 3 independent experiments, unpaired t-test; *p < 0.05, **p < 0.01, ***p < 0.001). Scale bar: 200 μm

To investigate the sensitization effects of HIT-4 on gemcitabine-resistance GBC cells to PARP inhibition in vivo, we established NOZ-R and GBC-SD-R subcutaneous xenograft models and administered vehicle, HIT-4, Olaparib, or their combination. As shown in Fig. 6A and Supplementary Fig. 6A-B, the combination treatment resulted in the most pronounced tumor growth inhibition, evidenced by significantly reduced tumor volumes and weights after 42 days, compared to either monotherapy. Importantly, no significant changes in body weight were observed among the treated groups (Supplementary Fig. 6 C), indicating a favorable toxicity profile and general tolerability of the regimen. Kaplan-Meier analysis further demonstrated that combined treatment significantly extended overall survival relative to control and single-agent arms (Supplementary Fig. 6D), suggesting a sustained therapeutic benefit. To elucidate mechanistic concordance with in vitro findings, we performed histopathological analyses of E2F8 and its downstream effector RRM2. Consistent with prior data, E2F8 expression remained largely unaffected by treatment, whereas RRM2 levels were markedly diminished in the HIT-4 and combination groups based on IHC H-score analysis (Fig. 6B and Supplementary Fig. 6E), supporting RRM2 as a key functional mediator. In addition, cleaved caspase 3 staining revealed significantly elevated apoptosis in tumors receiving the combined therapy (Supplementary Fig. 6 F), and γ-H2AX immunofluorescence showed a substantial increase in DNA damage, indicating intensified genomic instability under dual inhibition (Fig. 6C).

Building on these observations, we next validated the therapeutic synergy of HIT-4 and PARP inhibition in clinically relevant patient-derived organoid (PDO) models. Six PDOs were established—three from primary gallbladder cancers and three from recurrent, gemcitabine-treated tumors—according to a published protocol [40]. As shown in Fig. 6D, the GBC-derived organoids exhibited characteristic glandular and cribriform architectures. Immunohistochemistry revealed that recurrent PDOs (PDO4-6) expressed markedly higher levels of E2F8 and RRM2 than the primary PDOs (PDO1-3) (Supplementary Fig. 6G), which was further confirmed by Western blot analysis **(**Fig. 6E**)**. Drug sensitivity assays demonstrated that recurrent PDOs displayed lower IC_50_ values for HIT-4, indicating greater susceptibility **(**Fig. 6F**)**. Furthermore, combined HIT-4 and Olaparib treatment significantly inhibited the growth of recurrent PDOs compared with monotherapy, whereas this effect was not observed in the primary PDOs **(**Fig. 6G-H**)**. Bliss synergy analysis confirmed a strong synergistic effect (Bliss score >10) specifically in recurrent PDOs (Supplementary Fig. 6H).

Collectively, these results demonstrate that HIT-4 effectively sensitizes chemoresistant GBC cells to Olaparib both in vivo and in PDO models through inhibition of RRM2 and potentiation of DNA damage response, resulting in superior therapeutic efficacy without apparent systemic toxicity. This highlights the potential of dual targeting of E2F8-RRM2 axis and PARP-mediated repair pathways as a promising combinatorial strategy for overcoming chemoresistance in GBC.

## Discussion

Gallbladder cancer remains a formidable clinical challenge due to its aggressive biological behavior, frequent late-stage diagnosis, and pronounced resistance to chemotherapy [2, 5]. Although gemcitabine-based regimens have long been considered the mainstay for advanced GBC, their efficacy is severely compromised by the rapid development of chemoresistance [4, 41]. The scarcity of effective alternatives and poor prognosis underscore the urgent need to elucidate the molecular underpinnings of resistance and to identify actionable targets that can resensitize tumor cells to existing therapeutics. In this study, we reveal a novel resistance mechanism in GBC driven by the E2F8-RRM2 signaling axis. Our data demonstrate that E2F8 is markedly upregulated in gemcitabine-resistant GBC cells and that is directly enhances the expression of RRM2, a key regulator of DNA synthesis and repair. This upregulation augments the DNA repair capacity of tumor cells, enabling them to withstand gemcitabine-induced DNA damage. Genetic depletion of E2F8 or pharmacological inhibition using the small molecule inhibitor HIT-4 significantly sensitized resistant GBC cells to PARP inhibitors, both in vitro and in vivo. These findings highlight the therapeutic promise of targeting the E2F8-RRM2 axis to overcome chemoresistance in GBC.

Although E2F8 has been implicated in tumor progression and therapy resistance across various cancer types [22, 23, 42], specific inhibitors directly targeting this transcription factor remain unavailable. While nature products such as manzamine A have been proposed as potential E2F8 modulators [43], directing binding evidence remains lacking. In our study, we employed high-throughput virtual screening (HTVS) to identify HIT-4 as a novel small molecule that directly targets the DNA-binding domain of E2F8, illustrating the feasibility of inhibiting transcription factor activity with small molecules. Nonetheless, further structural characterization - particularly co-crystallization studies - will be essential to validate the binding interface, elucidate the structural basis of inhibition, and guide future lead optimization.

Our data also underscore the broader relevance of the E2F8-RRM2 axis in regulating DNA damage response and chemoresistance. Given the central role of RRM2 in nucleotide metabolism and its established involvement in resistance to nucleoside analogs such as gemcitabine [34], this axis may represent a conserved mechanism of multidrug resistance in other malignancies. Further studies are warranted to determine whether E2F8-RRM2 signaling functions as a common pathway in drug resistance across cancer types, which would significantly expand the clinical applicability of E2F8-targeted strategies. In addition, elucidating the molecular mechanisms responsible for the drug resistance-induced upregulation of E2F8 will be important for a more comprehensive understanding of its role in therapy resistance. Although E2F8 expression was predominantly observed in the tumor epithelial cells, a weak stromal response was also detected. Therefore, further investigations into the potential involvement of the E2F8-RRM2 axis within the tumor microenvironment may provide additional mechanistic insights.

In parallel, our findings broaden the therapeutic potential of PARP inhibitors beyond *BRCA-*mutant tumors. The combinatorial use of PARP inhibition with E2F8-targeting compounds achieved marked synergy in our GBC models, suggesting that disruption of DNA repair signaling through orthogonal mechanisms may enhance tumor vulnerability. Given that PARP inhibitors have demonstrated efficacy in overcoming chemoresistance in prostate, pancreatic, and lung cancers [44, 45], further exploration of their use in combination with transcription factor inhibitors could define a new paradigm in precision oncology. Clinical validation through well-designed trials will be critical to establish the translational potential of such regimens.

In conclusion, this study provides compelling evidence that the E2F8-RRM2 axis is a key driver of gemcitabine resistance in GBC and establishes a rationale for therapeutic targeting of E2F8 in combination with PARP inhibitors. These findings lay the groundwork for the development of novel small-molecule inhibitors that disrupt transcriptional regulation at the DNA-binding interface and address the critical unmet need posed by chemoresistant gallbladder cancer. Ongoing efforts in structural biology and drug optimization will be pivotal to translate these discoveries into clinically viable therapies.

## Conclusion

This study identifies the E2F8-RRM2 axis as pivotal mechanism underlying gemcitabine resistance in gallbladder cancer and demonstrates that targeting E2F8 significantly enhances tumor sensitivity to PARP inhibition. Through integrated CRISPR KO screening, transcriptomic profiling, and small-molecule discovery, we establish E2F8 as a druggable transcription factor that promotes DNA repair and survival in chemoresistant cells. The novel inhibitor HIT-4 selectively disrupts the E2F8-DNA interaction, downregulates RRM2 expression, and synergizes with PARP inhibitors to induce DNA damage and apoptosis both in vitro and in vivo. These findings not only unveil a therapeutic vulnerability in refractory gallbladder cancer but also open new avenues for transcription factor-targeted drug development.

## Supplementary Information

Supplementary Material 1.

Supplementary Material 2.

Supplementary Material 3.

Supplementary Material 4.

Supplementary Material 5.

Supplementary Material 6.

Supplementary Material 7.

## Data Availability

All data that support the findings of this study are available from the corresponding author (H.L.), upon reasonable request. The raw data for RNA-sequence in this article has been deposited in the Genome Sequence Archive (GSA for Human) at the National Genomics Data Center [46], China National Center for Bioinformation/Beijing Institute of Genomics, Chinese Academy of Sciences (Accession No. HRA014135).

## Abbreviations

GBC: Gallbladder cancer
CRISPR: Clustered Regularly Interspaced Short Palindromic Repeats
sgRNA: Single-guide RNA
HTVS: High-throughput virtual screening
DMSO: Dimethyl sulfoxide
PARP: Poly (ADP-ribose) polymerase
RRM2: Ribonucleotide reductase regulatory subunit M2
WB: Western blot
IHC: Immunohistochemistry
H&E: Hematoxylin and eosin
H-score: Histoscore
qPCR: Quantitative polymerase chain reaction
IC50: Half-maximal inhibitory concentration
siRNA: Small interfering RNA
shRNA: Short hairpin RNA
DSB: DNA double-strand break
