# Supplementary material for: Targeting E2F8 sensitizes gemcitabine-resistant gallbladder cancer to PARP inhibitors by disrupting RRM2-driven DNA repair
Source: J Exp Clin Cancer Res. 2025 Dec 14;45:19. doi: 10.1186/s13046-025-03586-2 (PMC12822184; doi:10.1186/s13046-025-03586-2)
Supplement: Supplementary file 7 — Supplementary Material 7. [file 13046_2025_3586_MOESM7_ESM.docx]

**Supplementary Information**

**Targeting E2F8 sensitizes gemcitabine-resistant gallbladder cancer to PARP inhibitors by disrupting RRM2-driven DNA repair**

**Contents:**

**Supplementary Figure 1-6**

**
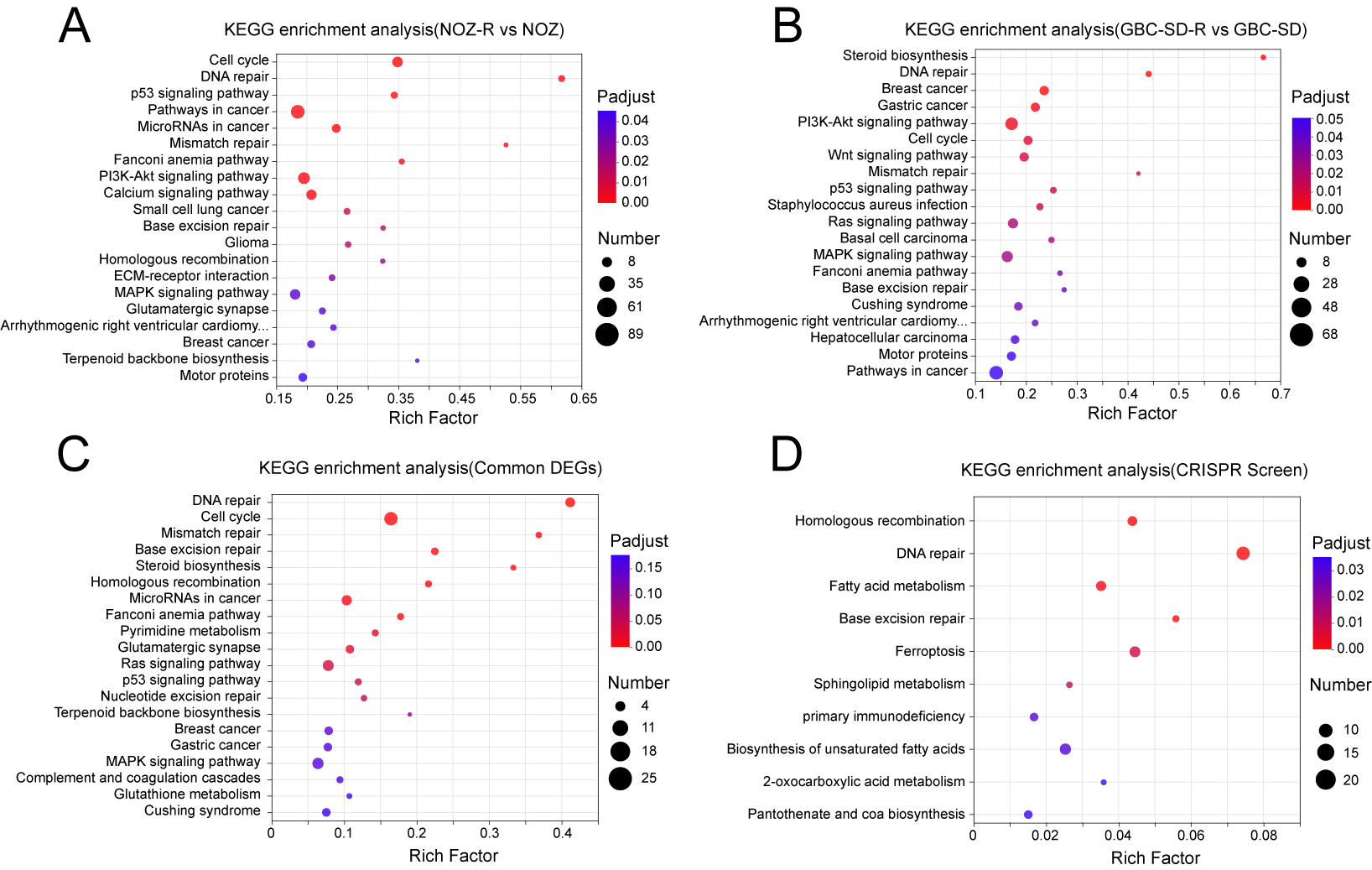
**

**Supplementary Figure 1. KEGG pathway enrichment analysis of differentially expressed genes (DEGs).**

(A) KEGG pathway enrichment analysis of DEGs identified between NOZ-R and NOZ cells. The Rich factor indicates the degree of enrichment for each pathway, with pathways listed on the Y-axis.

(B) Enriched KEGG pathways of DEGs identified between GBC-SD-R and GBC-SD cells.

(C) KEGG pathway enrichment analysis of common DEGs shared by NOZ-R vs NOZ and GBC-SD-R vs GBC-SD comparisons.

(D) KEGG pathway enrichment analysis of candidate genes identified from the CRISPR-Cas9 screening.

**
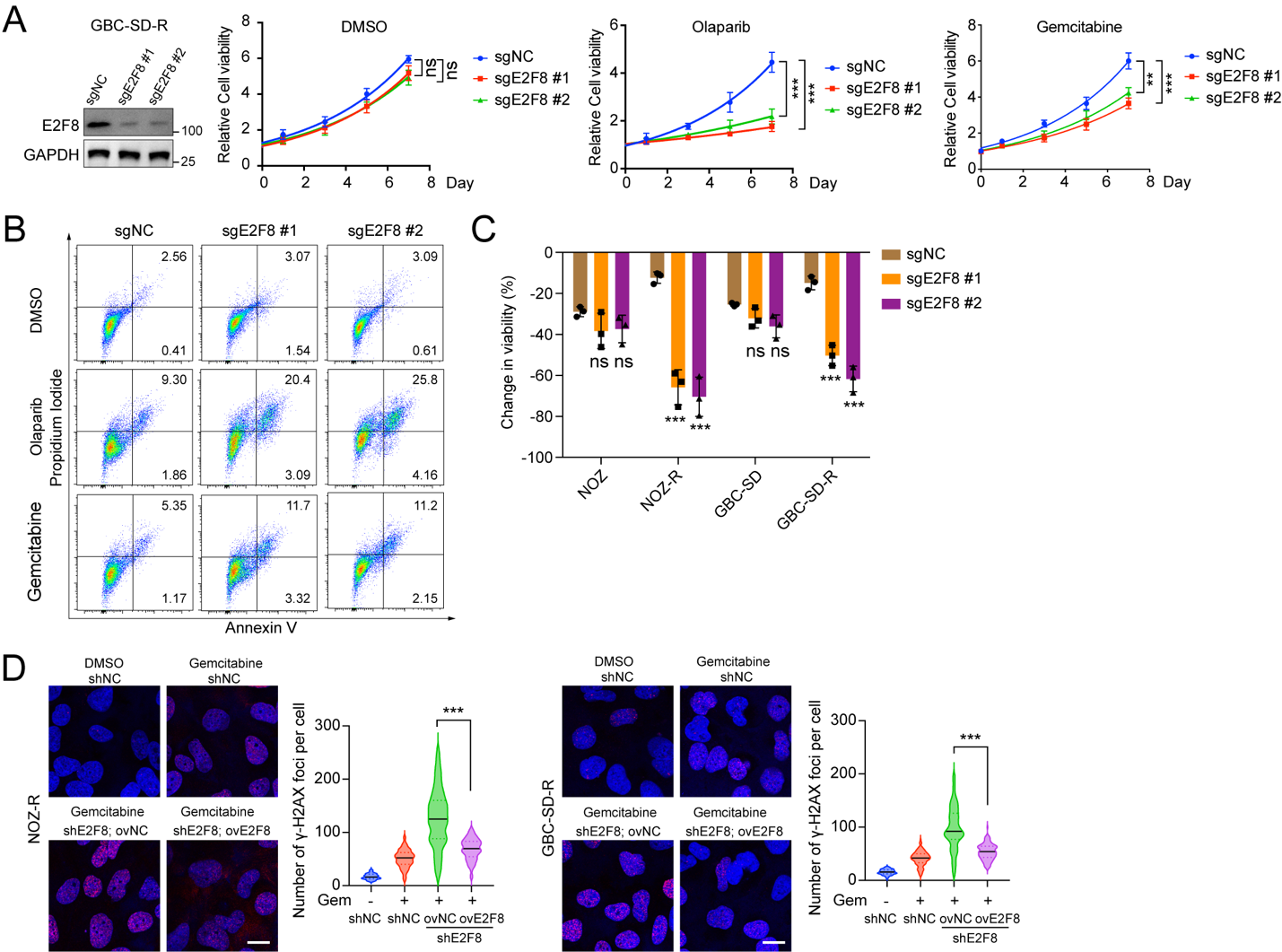
**

**Supplementary Figure 2. Depletion of E2F8 sensitizes resistant gallbladder cancer cells to PARP inhibition.**

(A) Knockdown of E2F8 enhances sensitivity to Olaparib and gemcitbaine in GBC-SD-R cells. Cells were transduced with control sgRNA (sgNC) or two independent sgRNAs targeting E2F8 (sgE2F8 #1 and sgE2F8 #2), then treated with DMSO, Olaparib (1 µM), or gemcitabine (0.1 µM). Western blot was performed to validate the efficiency of E2F8 knockdown. Data are shown as mean ± SD from three independent experiments (two-tailed t-test; ***p* < 0.01, ****p* < 0.001; ns, not significant).

(B) Apoptosis analysis of GBC-SD-R cells transduced with sgNC or sgE2F8 (#1 and #2), followed by treatment with DMSO, Olaparib (1 µM), or gemcitabine (0.1 µM) for three days. Quantification results for apoptotic cells are shown in Figure 2C.

(C) Cell viability assays were conducted on parental (NOZ, GBC-SD) and resistant (NOZ-R, GBC-SD-R) cells after E2F8 knockdown and subsequent treatment with Olaparib (1 µM) for 5 days. Data are presented as mean ± SD from three independent experiments (t-test; *** *p* < 0.001; ns, not significant).

(D) Immunofluorescence analysis and quantification of γ-H2AX foci per nucleus in NOZ-R (left) and GBC-SD-R (right) cells stably expressing E2F8-targeting shRNA (shE2F8), control shRNA (shNC), or E2F8-overexpressing cells with concurrent knockdown. Cells were pre-treated with DMSO or gemcitabine (0.5 µM) for 30 minutes, then fixed 4 hours post-treatment. γ-H2AX foci were quantified using Image-Pro Plus software. Scatter dot plots represent mean ± SD (n= 3 independent experiments, unpaired t-test; ****p* < 0.001).

**
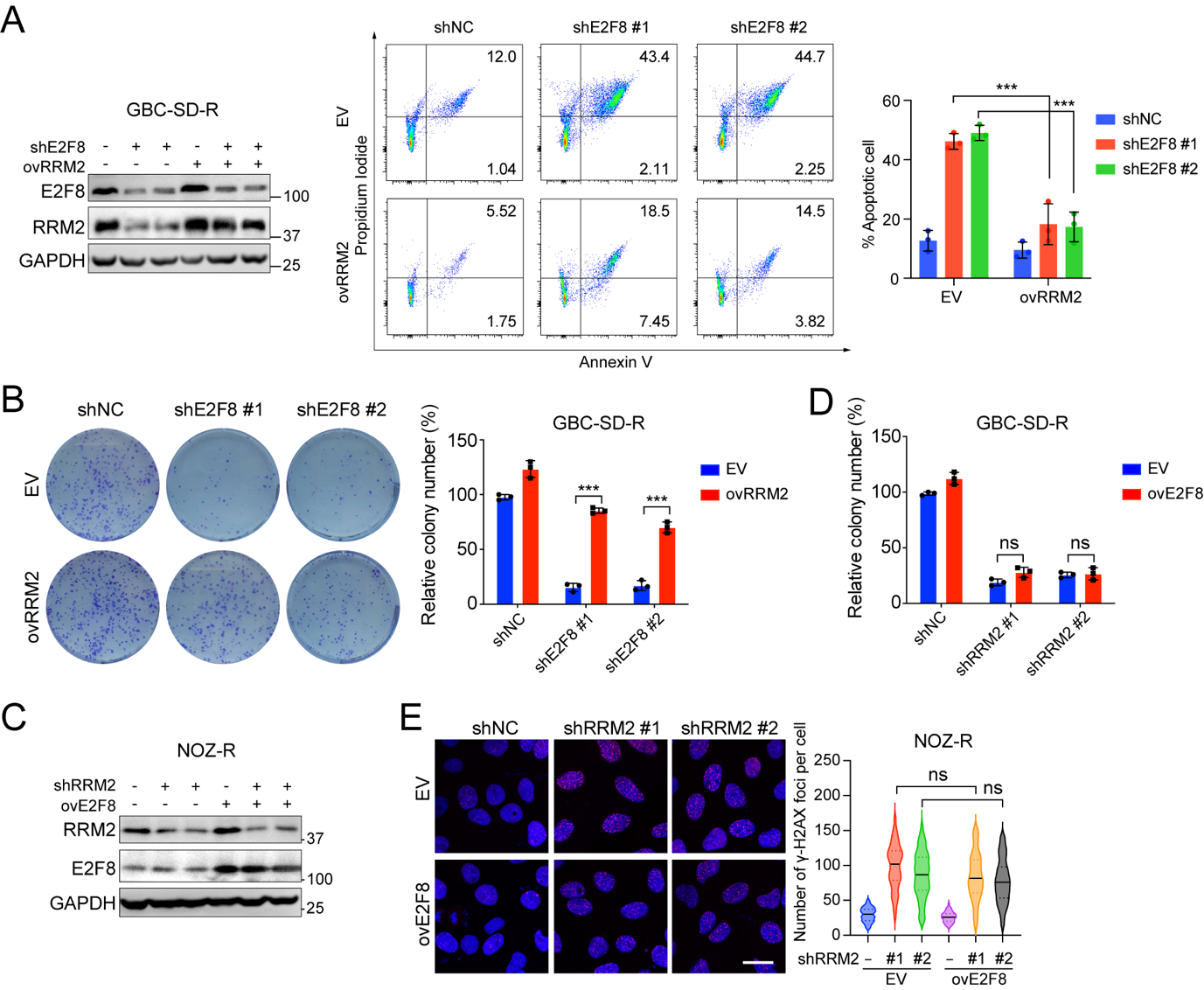
**

**Supplementary Figure 3. RRM2 is essential for E2F8-mediated regulation of sensitivity to PARP inhibitors.**

(A) Knockdown of E2F8 enhances sensitivity to Olaparib (1 µM, 3 days) in GBC-SD-R cells, which is reversed by RRM2 overexpression. Apoptotic cells were quantified using Annexin V/PI staining followed by flow cytometry. Data are presented as mean ± SD from three independent experiments (t-test; ****p* < 0.001).

(B) Colony formation assays demonstrating that overexpression of RRM2 rescues impaired proliferation induced by E2F8 knockdown (shE2F8 #1 and #2) in GBC-SD-R cells.

(C) Western blot analysis validating knockdown of RRM2 and overexpression of E2F8 in NOZ-R cells, corresponding to experiments presented in Figure 3J.

(D) Colony formation assays in shNC and shRRM2 (#1 and #2) GBC-SD-R cells, and in RRM2-knockdown cells overexpressing E2F8, demonstrating that E2F8 overexpression does not rescue proliferation defects caused by RRM2 depletion.

(E) γ-H2AX foci formation assay in NOZ-R cells treated with Olaparib (1 µM, 4 hours). RRM2 knockdown (#1 and #2) increases DNA damage, which is not mitigated by E2F8 overexpression. Quantification results are displayed as scatter dot plots (mean ± SD; n= 3 independent experiments; unpaired t-test; *** *p* < 0.001).

**
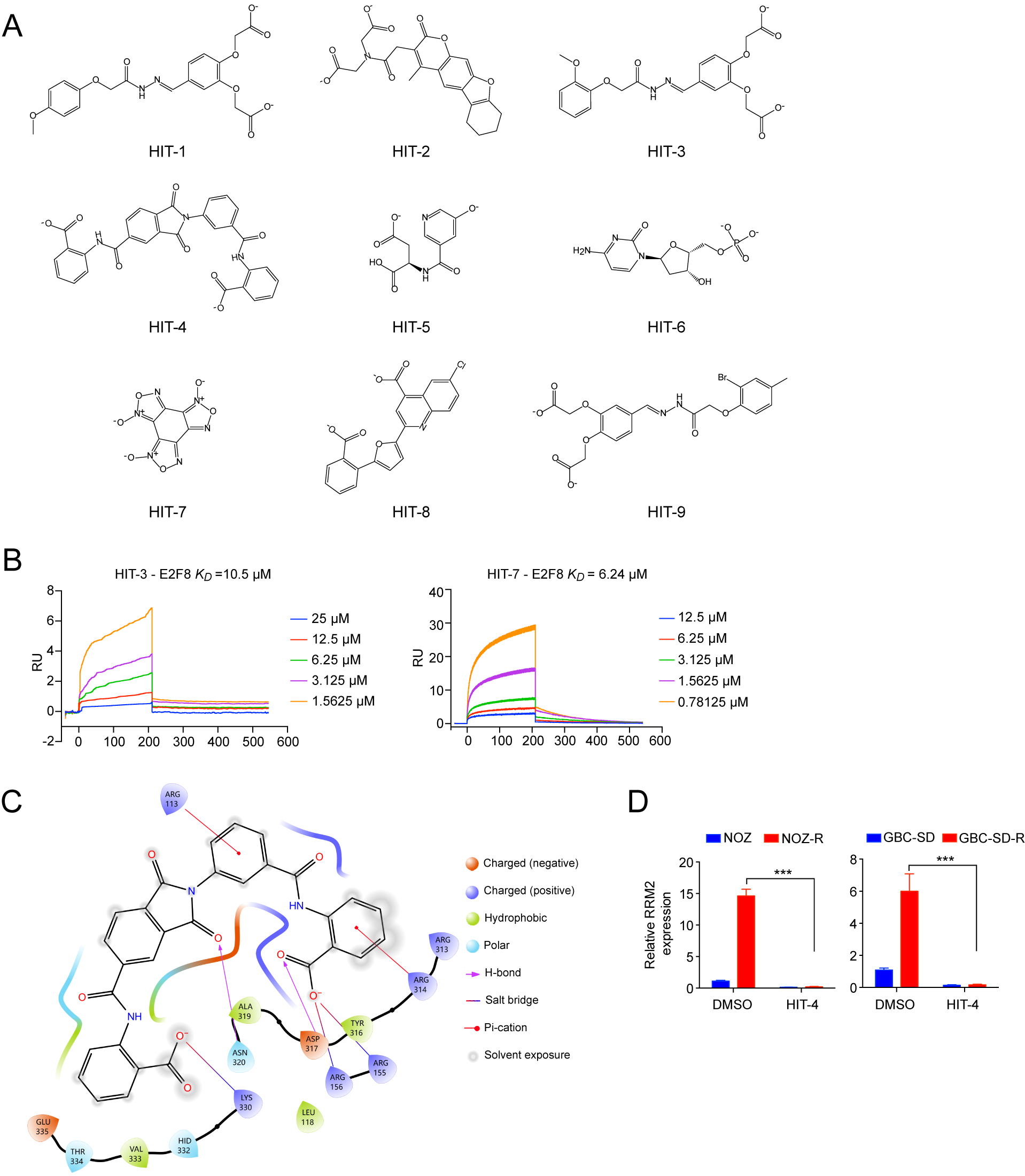
**

**Supplementary Figure 4. Structural and functional characterization of top candidate compounds.**

(A) Chemical structures of the 9 candidate compounds (HIT-1 to HIT-9).

(B) SPR results depicting the interaction between E2F8 and selected small molecules (HIT-3 and HIT-7). E2F8 protein was immobilized on a CM5 chip, and binding affinity constants were calculated using Biacore 8K evaluation software fitted to a 1:1 steady-state affinity model.

(C) Two-dimensional interaction schematic illustrating key contacts between HIT-4 and E2F8 (PDB ID: 4YO2).

(D) RT-PCR analysis of RRM2 mRNA levels in NOZ/NOZ-R and GBC-SD/GBC-SD-R cells treated with HIT-4 (1 µM) for 6 hours. Data are presented as mean ± SD from three independent experiments (t-test; ****p* <0.001).

**
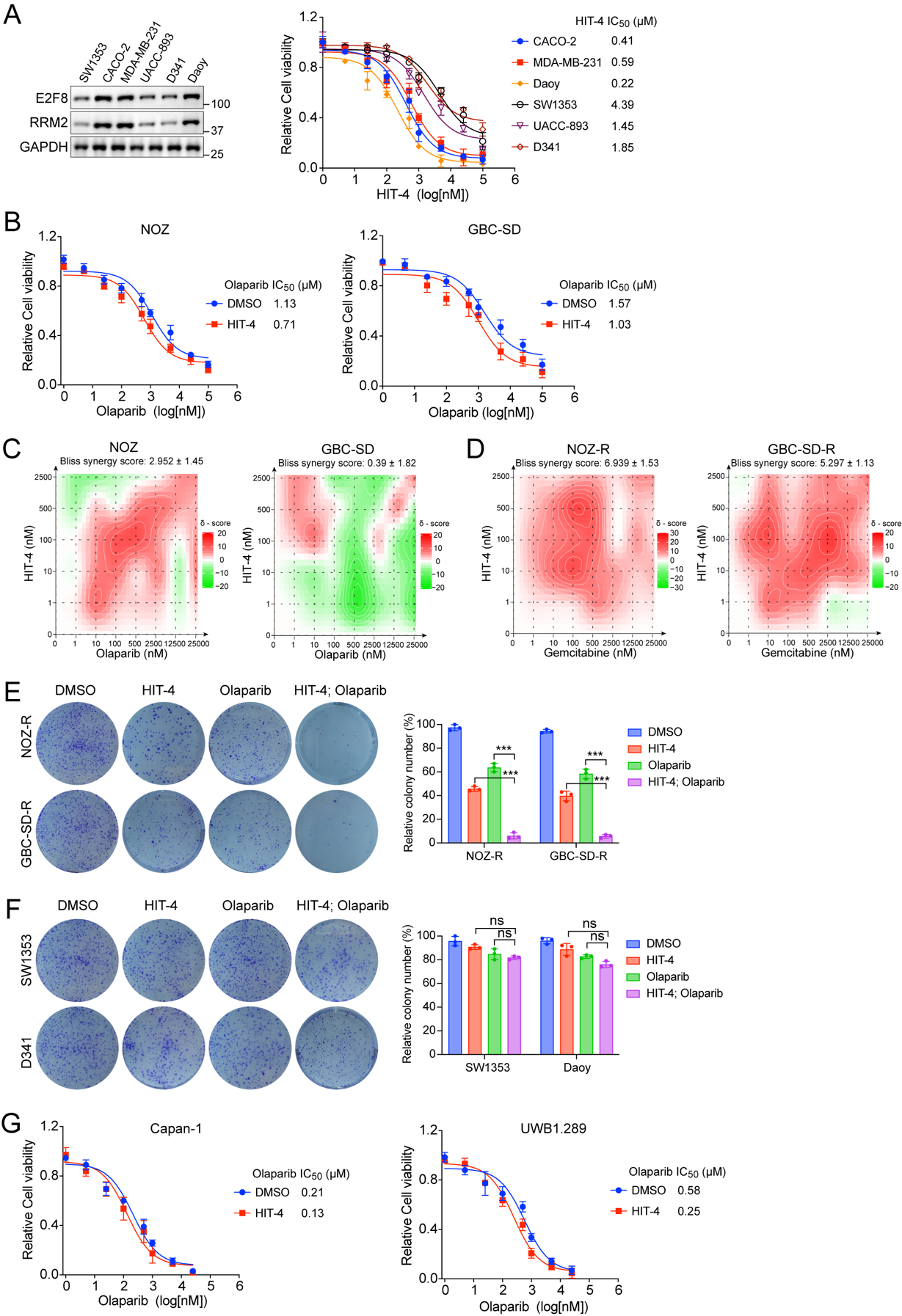
**

**Supplementary Figure 5. Evaluation of HIT-4 efficacy and synergy with Olaparib across diverse E2F8-expressing cancer cell lines.**

(A) Western blot analysis depicting E2F8 and RRM2 protein expression levels across multiple cancer cell lines (left). Dose-response curves showing cell viability following HIT-4 treatment for 5 days, measured using the CellTiter-Glo assay. Data are presented as mean ± SD from three independent experiments.

(B) Dose-response curves illustrating Olaparib sensitivity in NOZ and GBC-SD cells treated with DMSO or HIT-4 (1 µM), evaluated by CellTiter-Glo.

(C) Bliss synergy analysis for HIT-4 and Olaparib combination treatment in NOZ and GBC-SD cells. Cells were treated with increasing concentrations of HIT-4, Olaparib, or their combination for 96 hours.

(D) Bliss synergy maps showing the combinatorial effects of HIT-4 and gemcitabine in NOZ-R and GBC-SD-R cells after 96 hours of treatment. Bliss synergy scores > 10 indicate strong synergistic effects.

(E) Colony formation assays demonstrating decreased clonogenic capacity in NOZ-R and GBC-SD-R cells treated with HIT-4, Olaparib, or their combination.

(F) Colony formation assays in E2F8-low expressing SW1353 and Daoy cells following treatment with HIT-4 (1 µM), Olaparib (1 µM), or their combination for 2 weeks. Data represent mean ± SD (two-way ANOVA; ****p* < 0.001).

(G) Dose-response curves of Capan-1 and UBW1.289 cells treated with Olaparib in the presence of either DMSO or HIT-4 (1 µM). Cell viability was measured by CellTiter-Glo assay.

**
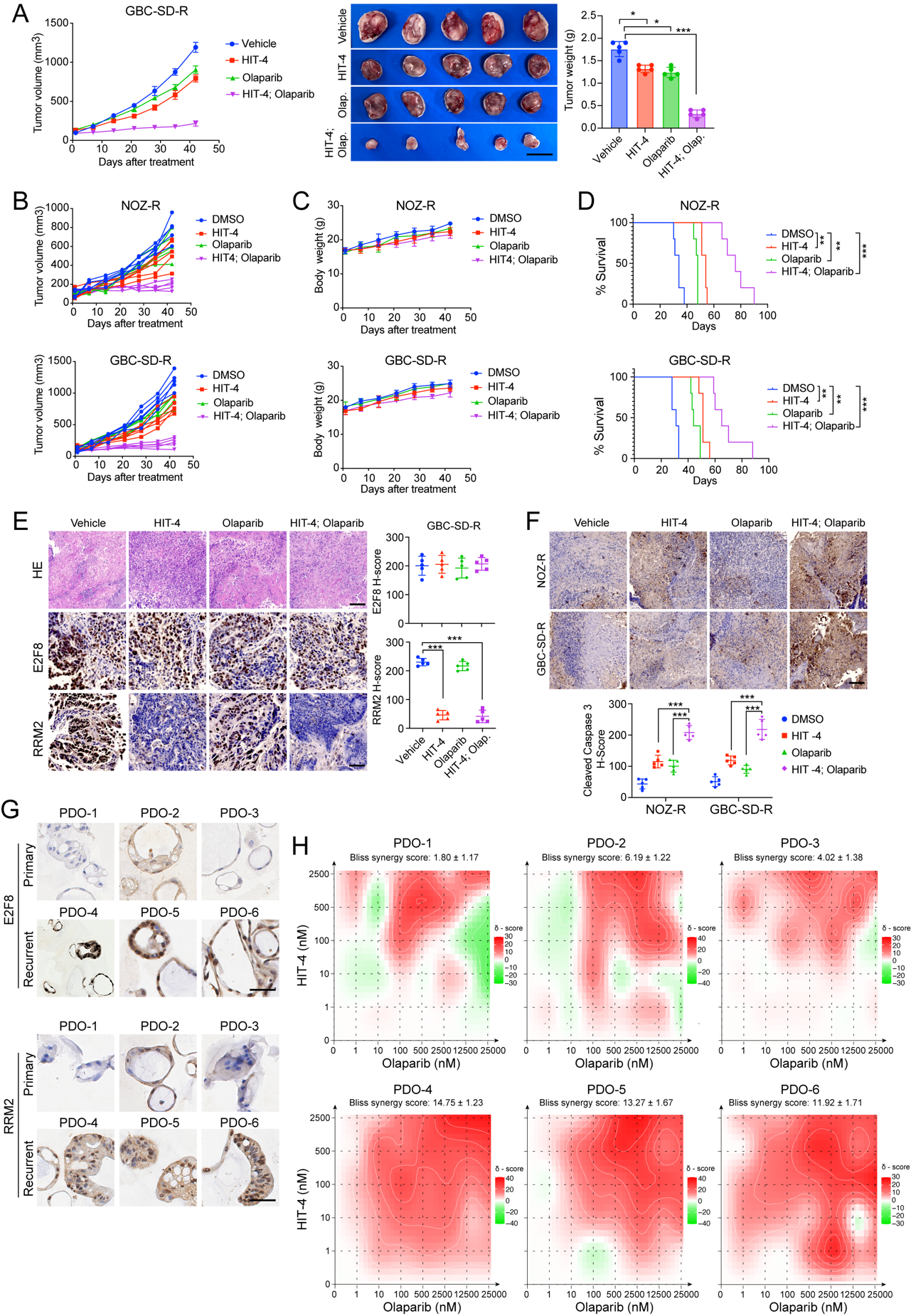
**

**Supplementary Figure 6. *In vivo* efficacy of HIT-4 combined with Olaparib.**

(A) Combination treatment with HIT-4 and Olaparib significantly reduces tumor growth in GBC-SD-R subcutaneous xenograft models. Cells were injected into the BALB/c nude mice, followed by random assignment to receive vehicle, HIT-4 (10 mg/kg), Olaparib (50 mg/kg), or the combination. Tumor volumes were measured weekly, and tumor were harvested and weighed after 42 days of treatment. Data are presented as mean ± SD (two-way ANOVA; **p* < 0.05, ****p* < 0.001). Scale bar: 1 cm.

(B) Individual tumor growth curves for each mouse in NOZ-R and GBC-SD-R xenograft models treated with vehicle, HIT-4, Olaparib, or their combination.

(C) Body weight changes in mice treated with vehicle, HIT-4, Olaparib, or their combination throughout the 42-day treatment period (n = 5 mice/group).

(D) Kaplan-Meier survival analysis of NOZ-R and GBC-SD-R xenografts-bearing mice treated with vehicle, HIT-4, Olaparib, or combination therapy (n = 5 mice/group). Mice were euthanized when tumor volume exceeded 1,000 mm^3^. Statistical significance was determined using log-rank (Mantel-Cox) tests.

(E) H&E and Immunohistochemical staining for E2F8 and RRM2 in GBC-SD-R subcutaneous tumor tissues. H-scores were quantified from tumors of five individual mice per group. Data are presented as mean ± SD (two-way ANOVA; ****p* < 0.001). Scale bar: 100 µm.

(F) Representative immunohistochemical staining for the apoptosis marker cleaved caspase-3 in NOZ-R and GBC-SD-R xenografts treated with vehicle, HIT-4, Olaparib, or their combination. H-scores were quantified from tumors of 5 individual mice per treatment group. Statistical significance was evaluated using two-way ANOVA. Scale bar, 100 µm.

(G) Representative IHC staining for E2F8 and RRM2 in PDOs derived from primary (PDO1-3) and recurrent (PDO4-6) tumors. Scale bar, 50 µm.

(H) Bliss synergy maps showing the combinatorial effects of HIT-4 and Olaparib in PDOs cells after 96 hours of treatment. Bliss synergy scores > 10 indicate strong synergistic effects.
